# Re-Ranking Sequencing Variants in the Post-GWAS Era for Accurate Causal Variant Identification

**DOI:** 10.1371/journal.pgen.1003609

**Published:** 2013-08-08

**Authors:** Laura L. Faye, Mitchell J. Machiela, Peter Kraft, Shelley B. Bull, Lei Sun

**Affiliations:** 1 Division of Biostatistics, Dalla Lana School of Public Health, University of Toronto, Toronto, Ontario, Canada; 2 Samuel Lunenfeld Research Institute, Prosserman Centre for Health Research, Mount Sinai Hospital, Toronto, Ontario, Canada; 3 Program in Molecular and Genetic Epidemiology, Department of Epidemiology, Harvard School of Public Health, Boston, Massachusetts, United States of America; 4 Department of Statistical Sciences, University of Toronto, Toronto, Ontario, Canada; Wellcome Trust Sanger Institute, United Kingdom

## Abstract

Next generation sequencing has dramatically increased our ability to localize disease-causing variants by providing base-pair level information at costs increasingly feasible for the large sample sizes required to detect complex-trait associations. Yet, identification of causal variants within an established region of association remains a challenge. Counter-intuitively, certain factors that increase power to detect an associated region can decrease power to localize the causal variant. First, combining GWAS with imputation or low coverage sequencing to achieve the large sample sizes required for high power can have the unintended effect of producing differential genotyping error among SNPs. This tends to bias the relative evidence for association toward better genotyped SNPs. Second, re-use of GWAS data for fine-mapping exploits previous findings to ensure genome-wide significance in GWAS-associated regions. However, using GWAS findings to inform fine-mapping analysis can bias evidence away from the causal SNP toward the tag SNP and SNPs in high LD with the tag. Together these factors can reduce power to localize the causal SNP by more than half. Other strategies commonly employed to increase power to detect association, namely increasing sample size and using higher density genotyping arrays, can, in certain common scenarios, actually exacerbate these effects and further decrease power to localize causal variants. We develop a re-ranking procedure that accounts for these adverse effects and substantially improves the accuracy of causal SNP identification, often doubling the probability that the causal SNP is top-ranked. Application to the NCI BPC3 aggressive prostate cancer GWAS with imputation meta-analysis identified a new top SNP at 2 of 3 associated loci and several additional possible causal SNPs at these loci that may have otherwise been overlooked. This method is simple to implement using R scripts provided on the author's website.

## Author Summary

As next-generation sequencing (NGS) costs continue to fall and genome-wide association study (GWAS) platform coverage improves, the human genetics community is positioned to identify potentially causal variants. However, current NGS or imputation-based studies of either the whole genome or regions previously identified by GWAS have not yet been very successful in identifying causal variants. A major hurdle is the development of methods to distinguish disease-causing variants from their highly-correlated proxies within an associated region. We show that various common factors, such as differential sequencing or imputation accuracy rates and linkage disequilibrium patterns, with or without GWAS-informed region selection, can substantially decrease the probability of identifying the correct causal SNP, often by more than half. We then describe a novel and easy-to-implement re-ranking procedure that can double the probability that the causal SNP is top-ranked in many settings. Application to the NCI Breast and Prostate Cancer (BPC3) Cohort Consortium aggressive prostate cancer data identified new top SNPs within two associated loci previously established via GWAS, as well as several additional possible causal SNPs that had been previously overlooked.

## Introduction

The challenges of precise identification of disease-causing variants underlying GWAS signals have recently received much attention [1]–[3]. For post-GWAS statistical analysis that aims to accurately identify potentially causal variants, a major hurdle is the development of methods to distinguish disease-causing variants from their highly-correlated proxies. While GWAS-era statistical methods focused on identifying associated regions via tag SNPs at the coarse scale of GWAS arrays, next generation sequencing (NGS) technology offers the capability to not only detect associated regions, but to distinguish the causal SNPs within these associated regions. Here we make a distinction between ranking SNPs across the genome to identify an associated region, and ranking to pinpoint the potential causal variant *within* an associated region. Identifying an associated region requires that trait-associated SNPs be ranked above null SNPs, while identifying the causal variant requires that, among associated SNPs, associations due to causality are ranked above indirect associations due to other factors, e.g. linkage disequilibrium (LD). GWAS and imputation studies typically report the top-ranked SNP for each associated locus, and follow-up studies typically attempt replication for these top-ranked SNPs (for further discussion of ranking see Text S1).

Zaitlen *et al* [4] proposed a measure of performance for sequencing and fine mapping analysis, their localization success rate metric is the probability that the causal SNP has the top-ranked test statistic within an associated region. When multiple SNPs are in high LD, the localization success rate drops dramatically [5]. Udler *et al* (2010) investigated the difficulty in overcoming the stochastic effect of high LD among causal and non-causal SNPs [5]. The sample size required to distinguish the causal SNP can be 1 to 4 times the size required to detect the association at genome-wide significance. Zaitlen *et al* [4] showed that this problem could be overcome through joint analysis of samples from carefully selected populations with differing LD structure. Although candidate causal SNPs will require further bioinformatic or functional study to ultimately delineate potential causal mechanisms, optimized study design and analysis can point to the best possible candidate causal SNP(s) and help develop testable hypotheses about biological mechanisms.

Studies of complex traits now underway are leveraging the cost efficiency of integrating GWAS, low- and high-coverage sequencing, and imputation to achieve sample sizes in the tens of thousands [6], [7]. For example, the Genetics of Type 2 Diabetes (GoT2D) study is combining low and high-coverage sequencing with 2.5M-SNP GWAS genotyping and imputation to achieve a total sample size of over 28,000 [8]. Sequencing the GWAS sample exploits the GWAS findings to ensure that an association signal is present at the genome-wide level and eliminates the cost of recruiting new individuals. Analysis of sequenced and imputed SNPs (post-GWAS data) can thus be informed by previous GWAS results, allowing a prioritized use of post-GWAS data in fine-mapping regions surrounding significant GWAS tag SNPs [9]–[11]. Selection of associated regions for further studies can also be based on combined GWAS and post-GWAS criteria [12], [13]. For example, the WTCCC [13] required a marginally significant (p-value<10^−4^) GWAS SNP to support the evidence at a genome-wide significant imputed SNP. However, these strategies lead to two important issues that have received little attention in the context of causal SNP identification: (1) the effect of the re-use of successful GWAS data and (2) the effect of genotyping error rates that differ between sequenced or imputed SNPs.

The re-use of GWAS data that had contributed to the identification of an associated region for post-GWAS analysis can adversely affect accurate causal SNP identification. For example, the simulation study of Wiltshire *et al* [14] showed that when a significant GWAS tag SNP is followed up by sequencing in the same sample, the tag SNP is in fact ranked higher than the true causal SNP 30% to 63% of the time, depending on the genetic model and effect size. When a GWAS tag SNP is selected based on small p-value, the magnitude of the association at the tag tends to be over-estimated; this form of selection bias is also known as the winner's curse [15]–[19]. To a variable extent, depending on the LD pattern, this selection bias is carried over from the GWAS tag to post-GWAS sequenced or imputed SNPs [20]. While this earlier work empirically demonstrated the effect of selection for a significant GWAS tag SNP on the causal SNP, no work to date explores whether it also affects the rank of the causal SNP among all neighboring SNPs within an associated region, and if so how to correct for the bias.

High error rates and differences in error rates, due to differences in coverage, read length and depth, minor allele frequency (MAF), GC content, local sequence structure, and other sequence-specific factors, are common to NGS SNPs and are well-recognized obstacles to analysis [21]–[29]. Error rates for low-read-depth sequencing studies are estimated to be 1%–3% [22], [30], [31], and as little as 1% error can produce a large loss in power [27]. The strategy of low-coverage sequencing in a portion of GWAS samples has been used to discover sequencing variants and build a reference panel to drive imputation in the remaining samples, but the genotyping accuracy can be worse than if all individuals were sequenced [25]. The choice of lower-coverage design is also motivated by reports that low-coverage sequencing in a large sample, alone or combined with GWAS and imputation data, can achieve superior power to detect associations compared to high-coverage sequencing in a small sample with similar cost [25], [29], [32], [33]. However, whether the localization success rate of the causal variants responsible for these associations is similarly high has not yet been examined. High error rates that differ among SNPs also occur in high-coverage sequencing; for example, within targeted high-coverage regions, highly repetitive elements can be difficult to capture resulting in low accuracy for some SNPs [34].

Differential genotyping accuracy between studies has been shown to reduce power of meta-analysis in the imputation setting [35], and differential accuracy between cases and controls has been shown to cause confounding and elevated type I error [36], [37]. Accounting for differential genotyping accuracy in the association test can recover some of the lost power and reduce type I error [35], [36]. However, whether it affects our ability to distinguish causal SNPs from correlated SNPs, and how best to account for the effect of differential genotyping accuracy jointly for all SNPs (GWAS tagged, imputed or sequenced) is an open question.

In this report, we first demonstrate that:

1. Localization success rate decreases as the correlation between the tag and causal SNP increases.
2. Selection at the tag SNP exacerbates this problem by increasing the magnitude of the association evidence at the tag SNP itself and at other neighboring SNPs in higher LD with the tag relative to the causal SNP.
3. Differential genotyping or imputation error between SNPs further decreases localization success rate, with or without the tag selection.
4. This problem can be exacerbated by increasing sample size, if genotyping accuracy at the causal SNP is lower than at neighboring SNPs.

We develop an analytic description of how these factors influence the probability of localization success and evaluate this probability for a range of plausible parameter values. We then show how to properly adjust for the adverse effects of these factors with a re-ranking procedure. We evaluate the performance of the method with extensive simulation studies under a wide range of realistic scenarios, and we demonstrate the practical use of re-ranking with an application to the NCBI BPC3 aggressive prostate cancer GWAS with imputation [38].

## Materials and Methods

Suppose that *M* sequenced (or imputed) SNPs, *S_i_, i  = 1, …, M*, in the region surrounding a significant GWAS tag SNP *G* are ranked by the magnitude of their association statistics in order to identify the causal SNP *C*. Table 1 provides the notation for the various parameters and statistics used throughout the report. Briefly, *T_Si_* is the Wald test statistic at a sequenced SNP *S_i_*; 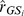 is the sample Pearson correlation coefficient between the GWAS/imputed/sequenced genotypes (most likely or fractional allele dosage) for SNPs *G* and *S_i_* (*r* ^2^ is the well-known pair-wise correlation measure of LD between two SNPs); 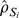 is the estimated correlation between the true genotype and the called genotype for a sequenced SNP *S_i_* (we use correlation as a measure of genotyping accuracy because of its simple interpretation in terms of power and genotyping quality; this quantity is provided by both MACH [24] and BEAGLE [39] software); 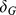 and 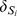 are proportions of samples with non-missing genotypes (termed call rates) at SNPs *G* and *S_i_*, respectively, and 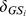 is the joint call rate, the proportion of samples with non-missing genotypes at both SNPs, and 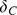 is the call rate at the causal SNP.

**Table 1:** Notation.

Sequenced or imputed SNPs indexed *i* 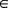 * 1 … M*	*S_1_ , … ,.S_M_*
Causal SNP	C
GWAS tag SNP	G
Test statistic at sequenced SNP *i*, causal SNP, GWAS tag SNP	*T_Si_*, *T_C_*, *T_G_*
Observed value of the test statistic at the tag SNP	*T_Gobs_*
Re-ranking statistic at sequenced SNP *i*	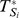
Correlation between:	
Actual genotypes of casual and tag, causal and sequenced SNP *i,*, tag and sequenced SNP *i*	*r_CG_, r_CSi_, r_GSi_*
Estimated genotypes for the tag and sequenced SNP *i*	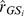
Actual genotype of the causal SNP and estimated genotype at sequenced SNP *i*	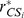
Call rate (1-missing data rate) at sequenced SNP *i*, tag SNP	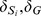
Joint call rate for tag SNP and sequenced SNP *i*	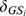
Correlation between actual and estimated genotypes at: sequenced SNP *i*, causal SNP, GWAS tag SNP	*ρ_Si_* ,*ρ_C_*, *ρ_G_*
Estimated correlation (sample correlation) of the above	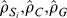
Tag selection bias (E[T_G_| threshold selection and ranking]-E[T_G_]), Bootstrap estimate of the bias	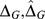
Genetic effect at the causal SNP, estimate	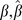
Standard deviation of the estimate at the causal SNP, estimate	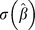 , 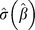
Sample size	*n*
Expected value of the test statistic at the causal SNP re-scaled for sample size	*μ_C_*
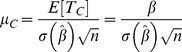	
Standard normal critical value at significance level 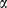 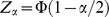	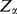
Standard normal cumulative distribution and density functions	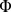 , 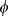

Let 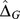 be an estimate of the selection bias in genetic effect estimation at the tag SNP *G* (described further below), that is the excess in the expected value of the test statistic 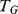 at the tag SNP *G* induced by selection based on its small p-value (or high rank). We call this phenomenon the *selection effect* (Δ*_G_* is zero if the region was not selected via a tag SNP that achieved the given significance or ranking criterion *in the same sample*). Our proposed re-ranking statistic for a sequenced SNP *S_i_* is
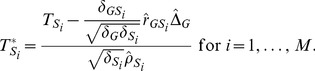
(1)
Equation (1) depends on the selection effect 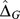, the tagging effect 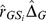, the genotyping accuracy effect 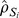 and scaling factors that depend on the call rates 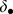. Justification for Equation (1) now follows in the remainder of this section. (Full details are provided in Text S2.)

Without loss of generality, let 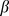
>0 be the genetic effect (e.g. the log odds ratio or the regression coefficient in the model relating the phenotype and genotype) at the causal SNP *C* which could be: one of the sequenced or imputed SNPs *S_i_*, *i  = 1,…, M*; the GWAS tag SNP *G* although this is unlikely; or neither if the genomic coverage was incomplete. Let the tag SNP *G* be coded such that the coded allele is positively correlated with the causal allele. Let 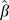 be the genetic effect estimate and 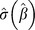 be the estimated standard deviation (SD) of the estimate from *n* observations. We assume that the distribution of the Wald test statistic at the causal SNP, 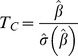 is approximately normal, 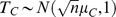, where 
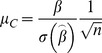
. The following also applies to test statistics that are asymptotically equivalent to the Wald test statistic.

Let 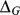 be the difference between the observed test statistic and its expected value,
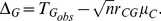
(2)Here 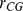 is the correlation between the genotypes of the causal *C* and the tag SNP *G*. (We assume that the tag is coded so that it is positively correlated with the risk allele of the causal SNP.) The value of 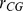 is unobserved and needed only in the theoretical formation of the problem not in the practical implementation, which we discuss later. The selection effect is most pronounced when there is low power at the tag SNP. (For discussion of this point see Text S3).

The conditional distribution of the test statistic *T_Si_* at the sequenced SNP *S_i_*, conditional on the value of the observed test statistic 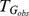 at the tag SNP *G*, is
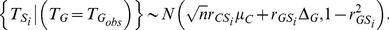
(3)Derivation of this distribution is detailed in Text S2. The first term, 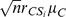, is the unconditional expected association signal at the sequencing SNP; the second term, 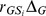, is the distortion due to the tag SNP selection propagated through correlation. Therefore, Δ*_G_*, the selection effect at the GWAS tag SNP *G* carries through to each sequenced SNP *S_i_* in proportion to the correlation 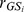 between *G* and *S_i_*. The combination of attenuation due to LD and upward selection bias at the tag, 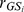 Δ*_G_*, distorts the association evidence so that SNPs in high LD with the tag are more likely to be top-ranked. We call this phenomenon the *tagging effect*, and use an estimate to remove bias from the conditional expected value of 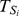 in (3).

Third, differential call rates among SNPs (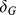, 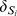 and 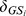) and estimated genotyping accuracy (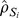 is the estimated and 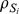 is the actual correlation between the called genotype and true genotype) of sequenced or imputed SNP *S_i_* appear in both the numerator and denominator of Equation (1). In the numerator, the tagging bias, 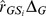, is scaled by a factor of 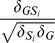 because correlation between the test statistics depends on the individual and joint call rates at the two SNPs (see Text S2 for derivation). The bias-corrected statistic in the numerator is scaled by 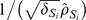 because
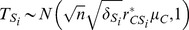
(4)where 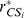 is the correlation between the genotype of the causal SNP and the *called or estimated* genotype of the sequenced SNP (in contrast to 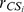, for the *true* genotype of the sequenced SNP). Assuming the probability of genotyping error is independent of the actual genotype, then 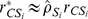
_._ It is clear that, without correction, smaller *ρ_Si_* (higher genotyping error) and smaller 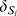 (higher missing data rate) tend to lower the probability that SNP *S_i_* would be top-ranked. We call this phenomenon the *genotyping accuracy effect*.

## Results

### Analytical study of the adverse effects of selection, tagging and genotyping accuracy on the localization success rate

To conceptually demonstrate the joint effects of selection, tagging and genotyping accuracy on the localization success rate (the probability that the causal SNP is topped ranked within an associated region), we first consider the simplified case of 2 SNPs, one causal (from sequencing or imputation) and one tag (from GWAS) with correlation between the two SNPs ranging from *r = *0.2 to 1 (from almost no LD to perfect LD). The inclusion of low LD value is motivated by the fact that correlation between the causal SNP and the best tag is often lower than expected. The coverage of GWAS platforms tends to be overestimated for both sequenced and imputed SNPs (see Text S4 for further discussion of this point). We assume that the MAFs of both SNPs are 0.12, the causal SNP has an additive odds ratio (OR) of 1.25, and selection at the tag SNP, if present, is based on its association test p-value<0.05 in a sample of 1000 cases and 1000 controls. Localization success rates (before applying the proposed re-ranking procedure) for all figures were computed based on Equations (2)–(3) and the equation in Text S3 and by numerically integrating over the following bivariate normal density function,
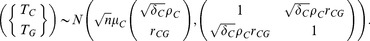
(5)


**Figure 1:**
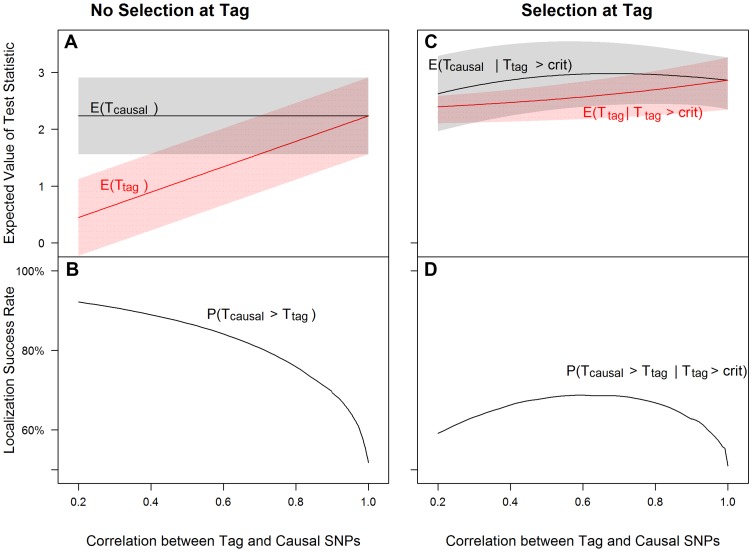
Tagging effect decreases localization success rates with or without the selection effect. The expected values of the association test statistics at a tag SNP (red) and the causal SNP (black), shading from 25^th^–75^th^ percentiles (**A, C**), and the localization success rates (**B, D**) for association studies (1000 cases and 1000 controls) of one causal SNP (MAF = 0.12; OR = 1.25; perfect genotyping accuracy) and one tag SNP (MAF = 0.12; in varying degree of correlation with the causal SNP, *r = *0.2 to 1; perfect genotyping accuracy) with no selection for significance at the tag SNP (**A, B**) or selection at the tag SNP requiring the test statistic *T_G_* to be significant with p-value<0.05 (**C, D**).

**Figure 2:**
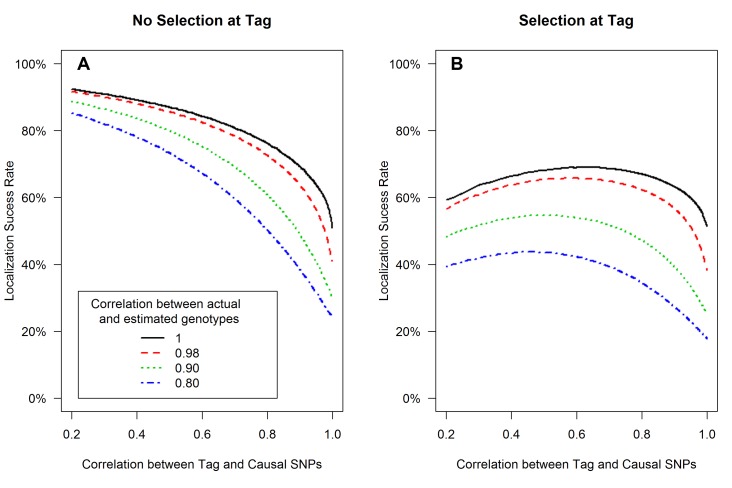
Low genotyping accuracy further reduces localization success rates with or without the selection effect. Localization success rates for association studies (1000 cases and 1000 controls) of one causal SNP (MAF = 0.12; OR = 1.25; *imperfect genotyping accuracy* due to sequencing or imputation errors resulting in correlation between the actual and estimated genotypes *ρ_C_ = *0.80 (blue dash-dotted) to 1 (black solid) and one tag SNP (MAF = 0.12; in varying degree of correlation with the causal SNP, *r_CG_ = *0.2 to 1 (X-axis); perfect genotyping accuracy with *ρ* ***_G_ = ***1) with no selection for significance at the tag SNP (**A**) or selection at the tag SNP requiring the test statistic *T_G_* to be significant with p-value<0.05 (**B**).

**Figure 3:**
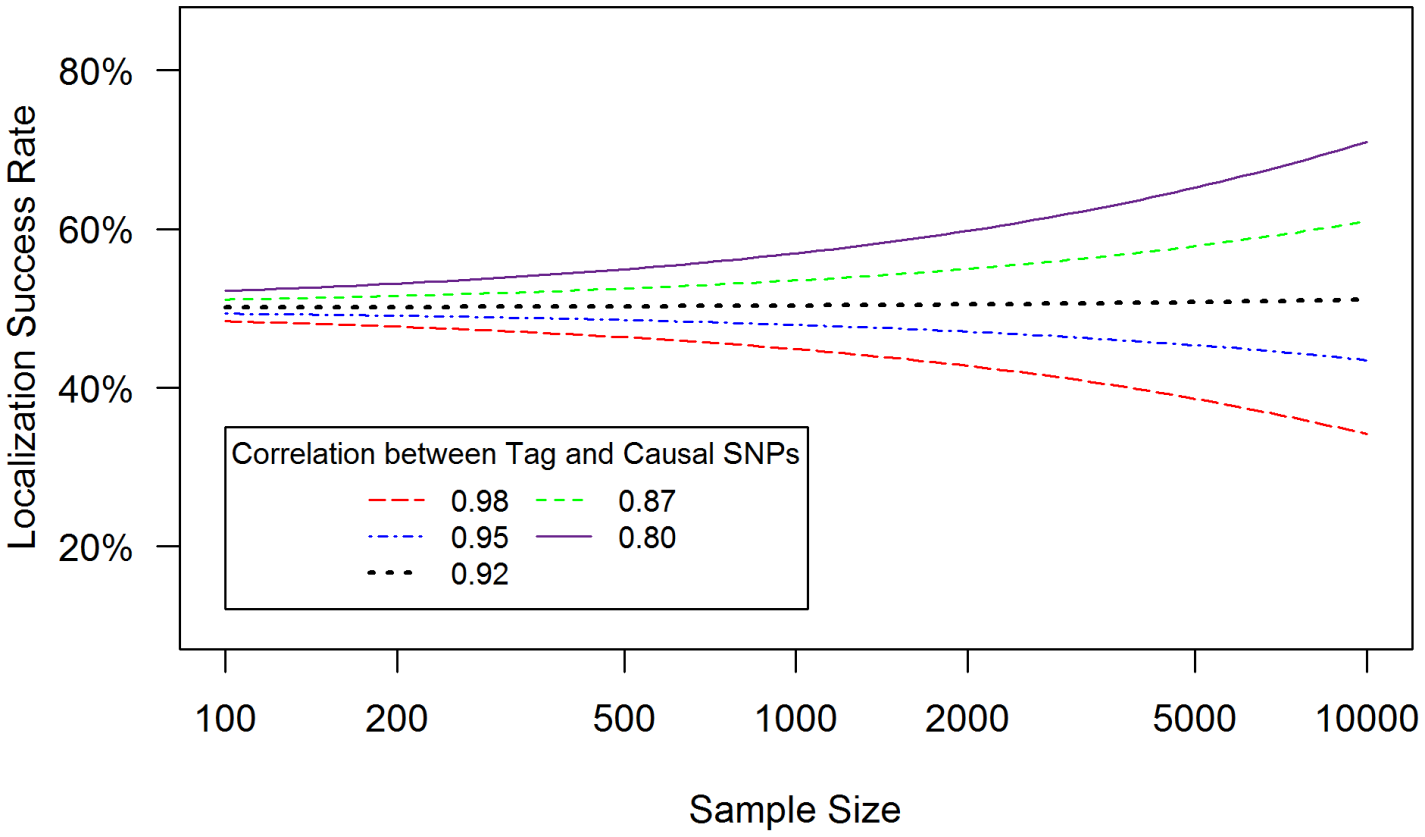
Well-tagged causal SNPs sequenced with low accuracy are unlikely to be correctly identified even as sample size increases. Localization success rates for association studies (sample size from 50∶50 cases∶controls to 5000∶5000 cases∶controls, X-axis) of one causal SNP (MAF = 0.12; OR = 1.25; *imperfect genotyping accuracy* due to sequencing or imputation errors resulting in correlation between the actual and estimated genotypes *ρ_C_ = 0.95*) and one tag SNP (MAF = 0.12; *in high correlation with the causal SNP, r_CG_* = 0.8 (purple solid) to 0.98 (red dashed); *100% genotyping accuracy with ρ* ***_G_*** *= 1*) with no selection for significance at the tag SNP.

Analytical evaluations of Equation (5) were used to generate Figures 1–3, which give insight into the relative influence of the tagging, selection, genotyping accuracy and sample size effects outlined in the Introduction and explicitly defined in Materials and Methods. We find similar patterns of influence for a rare SNP (MAF = 0.02, OR = 1.5; Figures S2, S4 and S6) and a higher frequency SNP (MAF = 0.25, OR = 1.25; Figures S3, S5 and S7), and when the number of non-causal SNPs increases (Figures S8, S9, S10).

#### (1) Tight linkage disequilibrium between SNPs can obscure the causal SNP (Figure 1)

Figure 1 left panel shows that as the correlation between tag and causal SNPs increases (X-axis), the expected association evidence at the tag, E[T_tag_], approaches E[T_causal_] (Figure 1A), resulting in a lower localization success rate (Figure 1B). As expected, increasing the number of non-causal SNPs in strong correlation with the causal SNP increases competition for the top rank and decreases the localization success rate (Figures S8, S9, S10). Increasing the number of non-causal SNPs in competition with the causal from 1 to 6 decreases the localization success rate from over 50% to less than 35% (bottom left panels of Figures 1 and S10).

#### (2) Selection at the tag SNP inflates the association evidence at the tag, increasing the probability that it out-ranks the causal SNP (Figure 1)

Figure 1 right panel shows that tag selection reduces the difference between the expected association evidence at the tag (E[T_tag_|T_tag_>crit]) and the causal (E[T_causal_|T_tag_>crit]), compared with no selection, regardless of the LD between the two SNPs (Figure 1C vs. 1A). Consequently, the localization success rate conditional on selection can be reduced by 25% as compared to the unconditional localization success rate (Figure 1D vs. Figure 1B). Results are similar for the rare SNP and more common SNP cases (Figures S2 and S3).

#### (3) Sequencing or imputation error decreases the localization success rate, with or without tag selection (Figure 2)

Low genotyping accuracy at the causal SNP reduces the expected value of it's test statistic, leading to decreased localization success rate (Figure 2). For example, if the tag SNP was genotyped with perfect accuracy (*ρ_G_* = 1), while the causal SNP was not, and if the genotype error at the causal SNP resulted in *ρ_C_* = 0.80 (the blue dash-dotted curve), then the localization success rate would be reduced by an additional 10%–30% as compared to perfect genotyping accuracy (the black solid curve). Results are similar for the rare SNP and more common SNP cases (Figures S4 and S5).

#### (4) Counter-intuitively, sample size can reduce localization success rate (Figure 3)

When the causal SNP is less accurately genotyped than one of its highly correlated proxies (i.e. *ρ_C_*<*ρ_G_* and *r_CG_* is large), the proxy SNP may capture the association better than the causal SNP. As a result, this proxy SNP will out-rank the causal SNP more than 50% of the time. In this case, the localization success rate would be less than 50%, and would decrease further as sample size increases (Figure 3). For example, if *ρ_C_* = 0.95, *ρ_G_* = 1 and *r_CG_* = 0.98 (red dashed line), the localization success rate drops from 47% to 26% as sample size increases from 100 to 10,000. Lower *ρ_c_* would lead to even lower localization success rates (results not shown). This pattern is similar for the rare SNP and more common SNP cases (Figures S6 and S7). We also note that, depending on the NGS experiment or the imputation parameters (e.g. the matching between the reference and imputation samples) for estimated genotype at the causal SNP *C*, the *ρ_C_* may not be lower bounded by the tagging *r_CG_*, which we discuss further in Text S4.

### Practical implementation of the post-GWAS re-ranking statistics

The above analytical results demonstrate the need to correct for the joint effects of selection, tagging and genotyping accuracy on the localization success rate. The practical implementation of the proposed re-ranking statistic in Equation (1) is as follows. The estimated selection bias 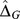 at the tag SNP *G* can be obtained using *BR-squared* that provides Bias-Reduced estimates via Bootstrap Resampling at the genome-wide level [40], [41]. (The original program, designed to provide estimates for the genetic effect *β*, has been modified slightly to provide estimates for the test statistic *T*; see software documentation on author's website for details.) The bootstrap estimator can be applied whether the region of interest was selected by rank or by p-value threshold. Unlike the threshold-based likelihood and Bayesian methods [42]–[46], the genome-wide bootstrap method incorporates information across the entire GWAS in order to account for the effects of LD and rank on the bias at each SNP. The values of the individual and joint call rates 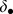 are available from the dataset, and genotype correlation 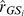 can be estimated from the sample. Correlation between the actual and estimated genotypes at a sequenced SNP 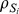 can be obtained from the mean posterior genotype (e.g. MACH ratio of variances estimate, [24]) or from the full genotype posterior probabilities (e.g. BEAGLE allelic *r^2^* estimate [39]). An R script that implements Equation (1) is available. The R script calls the BR2 software (http://www.utstat.toronto.edu/sun/Software/BR2/), which provides the essential quantity of 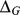 if the original GWAS dataset was used for fine-mapping.

### Simulation study design

We conducted extensive simulation studies to empirically evaluate the performance of the re-ranking method under five general scenarios (Table 2):

**Table 2:** Parameters and parameter values of the main simulation studies.

	Scenario 1	Scenario 2	Scenario 3	Scenario 4	Scenario 5
	Effect of selection, tagging, genotype accuracy	Effect of selection, genotype accuracy	Effect of genotype accuracy	Effect of multiple causal SNPs	Effect of missing data
Sample sequenced	Same as the Discovery sample, conditional on significance at the GWAS tag SNP (p<5×10^−7^)	Same as the Discovery sample, conditional on significance at any of the SNPs in the region (p<5×10^−7^)	Independent sample	Independent sample	Independent sample
# of GWAS tag SNPs in the region, *G*	1	1	1	1	1
# of post-GWAS SNPs (# of causal SNPs) in the region, *M*	10 (1)	10 (1)	10 (1)	11 (2)	10 (1)
OR of the causal SNP(s), β	1.5	2	2	2, 2	2
MAF of the tag and post-GWAS SNPs	4.8%	5%	5%	5%	5%
Correlation between the tag and causal SNP(s), *r*	0.78, 0.83, 0.85, 0.90, 0.93, 0.95, 1	0.95	0.95	0.80, 0.95 (*r* = 0.73 between the two causal SNPs)	0.95
Correlation between two adjacent non-causal post-GWA SNPs, *r*	0.975	0.975	0.975	0.975	0.975
Correlation between the actual and called genotypes for sequenced/imputed SNPs, *ρ_Si_*	0.82, 0.86, 0.90, 0.95, 0.97, 1	Same as S1	Same as S1	Same as S1	1
Call rates (1-missing data rate), 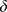	100%	100%	100%	100%	80%, 90%, 95%, 98%, 99% or 100%
Sample size, *n*	4901 (1963 cases and 2938 controls of the WTCCC T1D study)	2500, 5000, 7500 or 10,000 (equal cases and controls)	Same as S2	Same as S2	Same as S2
Simulation replicates for each configuration	300	800	800	800	800
Localization Success Rate	P(the causal SNP is top-ranked)	Same as S1	Same as S1	Defined for each of the 2 causal SNPs as P(the causal SNP ranks in top 2)	Same as S1

- **Scenario 1: GWAS used for discovery, and sequencing/imputation used for fine-mapping around GWAS “hits” using the same GWAS sample.** Scenario 1 is a GWAS-focused design based on the WTCCC Type 1 Diabetes substudy data. A significant region is identified by a significant GWAS tag SNP (p<5×10^−7^) and followed by fine-mapping with post-GWAS data (sequenced or imputed SNPs) in the region surrounding the tag SNP. The SNP with the largest test statistic in the region is selected as the best candidate causal SNP. Data is simulated as follows.
  1. **GWAS Data and Tag SNP:** In order to generate realistic data, we used the individual level genotypes from the WTCCC T1D sub-study as the GWAS data (1963 cases and 2938 controls); by fixing the genotypes we preserve realistic LD correlation structure over the entire genome. Among the reported WTCCC T1D significant regions, we randomly selected 12q24 109.82–111.49 Mb as the region of interest and designated rs11066410 (MAF 4.8%) as the GWAS tag SNP. In Scenario 1, the GWAS data are the WTCCC T1D substudy including GWAS genotyping data on all 4901 subjects. By fixing the genotypes we preserve realistic LD structure over the entire genome. We then simulate phenotype datasets that are significant at the tag SNP, rs11066410. Other tag SNPs in the region could be also significant, however in order to tease out the effect of tagging from other factors such as LD structure between sequencing SNPs in the region and MAF, we selected simulation datasets conditional on significance at rs11066410.
  2. **Sequencing/Imputation Data and Causal SNP:** We simulated sequencing data for the region of interest with 10 post-GWAS SNPs, among which one is the causal SNP with OR = 1.5. We varied the correlation between the tag and the causal SNP from *r* = 0.78 (causal not well tagged by the GWAS SNP) to 1 (the GWAS tag SNP is the causal, although this is an unlikely scenario). We introduced random error into post-GWAS SNP genotypes at per-allele rates 2%, 1.5%, 1%, 0.5%, 0.25% or 0%, so that the average *ρ_Si_* in each case was 0.82, 0.86, 0.90, 0.95, 0.97 or 1, respectively. Each sequencing SNP is in LD with the causal SNP as well. The correlation between the sequencing SNPs and the causal SNP ranges from 0.78 to 0.975.
  3. **Phenotype:** Phenotype datasets significant (p<5×10^−7^) at the GWAS tag SNP were simulated using a logistic model with causal SNP OR = 1.5.
- **Scenario 2: All GWAS and sequenced/imputed SNPs used for discovery and fine-mapping in the same dataset.** Here we assumed that all GWAS and post-GWAS SNPs are used to identify an associated region (p<5×10^−7^), and the most significant SNP in the region is then identified as the best candidate causal SNP. GWAS tag SNP data were simulated with MAF 5%. Sequencing data were simulated as described in Scenario 1 with parameter values detailed in Table 2. Phenotype datasets significant (p<5×10^−7^) at any GWAS or post-GWAS SNP, were simulated using a logistic model with causal SNP OR = 2.
- **Scenario 3: Discovery and fine-mapping using different datasets.** In this scenario, the region of interest was discovered in a previous study, while sequencing is performed in an independent dataset without conditioning on significance of the GWAS tag SNP in the independent dataset. Genotype and phenotype data were simulated as in Scenario 2, except that phenotype datasets were not selected for significance.
- **Scenario 4: Multiple causal SNPs.** To explore the effect of multiple causal SNPs, we re-considered scenario 3 but we assumed there are 11 fine-mapping sequenced/imputed SNPs, among which 2 are causal (both OR = 2).
- **Scenario 5: Missing data.** This scenario focuses on the effect of missing data (e.g. imperfect call rate). Genotype and phenotype data were simulated as in Scenario 3, except that genotyping accuracy was perfect. The missing rates were randomly assigned to each SNP so that the missing data proportion was between zero and twice the average error rate.

The parameter values in Table 2 were chosen to best reflect realistic scenarios. For example, in order to address realistic tagging, we examined the Affymetrix 5.0 chip and identified the SNP that best captured each significant WTCCC T1D GWAS SNP. The correlation between the two SNPs ranges from r = 0.79 to 1. For the range of genotyping accuracy, we note that in practice, the average sequencing *ρ* can vary substantially from study to study. For example, for low-coverage studies, it can vary from 0.63 to 0.99 depending on the coverage, MAF and sample size [25]. When low-coverage sequencing (4×) and imputation are combined, the average *ρ* can range from 0.89 to 0.99 depending on the reference panel size [24]. Sequencing *ρ* also depends on MAF; the same error rate in a lower MAF SNP results in a smaller *ρ*.

Even when the average *ρ* is high, SNP-level *ρ* can vary widely within a single study. Browning and Browning [39] found that imputation with a phased reference panel of 60 Hapmap CEU samples yielded a median *ρ* of 0.95, however individual *ρ* was less than 0.77 for 20% of the SNPs. We show that coverage rates can also vary widely between SNPs (Figure S1) by examining the 1000 Genomes low-coverage whole-genome pilot data from chromosome 1 in the CHB and JPT samples (Figure S1; October 2010 release; 1000 Genomes Project, 2010). We mimicked this variability in our simulations by randomly assigning each SNP in each dataset an error rate that ranged from zero to twice the overall average error rate. No random error however was introduced into the genotypes of the tag SNP (*ρ_G_* = 1), because GWAS genotyping has been estimated to be over 99.8% accurate [13], [47]. In order to ensure realistic correlation structure among post-GWAS sequencing/imputation SNPs, we examined all SNPs in the regions surrounding the WTCCC T1D significant SNPs using the HapMap3 dataset. The average correlation between adjacent SNPs in these regions was approximately 0.975.

### Simulation study results

One of the main findings of the simulation study is that GWAS-based region selection or moderate genotyping error can substantially reduce the probability of correctly identifying the causal SNP (Tables 3–4 and Tables S1, S2), consistent with that of the analytical study. For example, results detailed in Table S1 demonstrate that the combined tagging and genotyping accuracy effect can reduce the localization success rate by over 30%.

**Table 3:** Localization success rates for simulation Scenarios 1, 2, 3, 4.

		Average correlation between the actual and estimated genotypes of sequenced or imputed SNPs, *ρ_Si_*											
Correlation between the tag and causal SNPs, r		◂^---------------------^ Low-coverage Sequencing ^---------------------^▸								High-coverage Sequencing			
	Sample	0.82		0.86		0.90		0.95		0.97		1.00	
	size^b^	Naïve^c^	Re-ranked^d^	Naïve	Re-ranked	Naïve	Re-ranked	Naïve	Re-ranked	Naïve	Re-ranked	Naïve	Re-ranked
**Scenario 1** a													
0.78	4901	0.20	0.38	0.21	0.42	0.23	0.42	0.34	0.60	0.29	0.49	0.42	0.60
0.83	4901	0.12	0.35	0.16	0.41	0.20	0.47	0.26	0.52	0.34	0.54	0.48	0.64
0.85	4901	0.20	0.39	0.23	0.43	0.26	0.51	0.33	0.47	0.34	0.47	0.43	0.55
0.90	4901	0.09	0.32	0.15	0.43	0.18	0.45	0.31	0.56	0.34	0.43	0.40	0.57
0.93	4901	0.08	0.41	0.12	0.35	0.19	0.40	0.34	0.53	0.44	0.54	0.45	0.56
0.95	4901	0.11	0.32	0.09	0.31	0.21	0.42	0.23	0.33	0.32	0.42	0.42	0.54
Tag is causal^e^	4901	0.93	0.17	0.89	0.26	0.89	0.25	0.80	0.36	0.72	0.39	0.55	0.29
**Scenario 2** a													
0.95	2500	0.12	0.28	0.12	0.29	0.17	0.34	0.26	0.38	0.33	0.35	0.50	0.50
	5000	0.13	0.36	0.18	0.41	0.23	0.43	0.32	0.50	0.45	0.53	0.58	0.58
	7500	0.13	0.46	0.16	0.50	0.22	0.52	0.39	0.58	0.51	0.64	0.72	0.72
	10000	0.12	0.48	0.16	0.53	0.23	0.55	0.37	0.67	0.54	0.68	0.74	0.74
**Scenario 3** a													
0.95	2500	0.12	0.24	0.14	0.28	0.18	0.29	0.28	0.35	0.33	0.36	0.46	0.46
	5000	0.13	0.37	0.18	0.35	0.21	0.44	0.33	0.49	0.43	0.52	0.56	0.56
	7500	0.14	0.47	0.17	0.46	0.23	0.53	0.37	0.56	0.50	0.59	0.64	0.64
	10000	0.12	0.52	0.19	0.56	0.22	0.57	0.42	0.63	0.55	0.68	0.78	0.78
**Scenario 4** a													
0.80	2500	0.07	0.14	0.07	0.16	0.10	0.17	0.13	0.19	0.18	0.20	0.23	0.23
	5000	0.05	0.14	0.07	0.16	0.08	0.17	0.13	0.17	0.16	0.20	0.21	0.21
	7500	0.06	0.18	0.06	0.17	0.10	0.20	0.14	0.21	0.18	0.24	0.22	0.22
	10000	0.04	0.15	0.05	0.16	0.08	0.18	0.10	0.21	0.16	0.23	0.23	0.23
0.95	2500	0.04	0.15	0.07	0.15	0.07	0.16	0.11	0.18	0.14	0.18	0.24	0.24
	5000	0.04	0.13	0.04	0.14	0.05	0.18	0.10	0.17	0.13	0.18	0.20	0.20
	7500	0.04	0.18	0.04	0.20	0.05	0.20	0.12	0.23	0.18	0.25	0.25	0.25
	10000	0.03	0.16	0.03	0.16	0.04	0.16	0.09	0.19	0.12	0.19	0.20	0.20

a See Table 2 for details of the simulation models; scenario 4 has two causal loci.

b 1963 cases and 2938 controls for Scenario 1; equal number of cases and controls for Scenario 2,3,4.

c Naïve is standard ranking without correction for selection or genotyping error.

d Re-ranked is ranking by corrected statistic in Equation 1.

e In this simulation, the GWAS tag SNP is causal and all post-GWAS SNPs are non-causal.

**Table 4:** Localization success rates for simulation Scenarios 5^a^.

Correlation between the tag and causal SNPs, r		Call Rate ( = 1-Missing Data Rate)											
	Sample	0.80		0.90		0.95		0.98		0.99		1.00	
	size^b^	Naïve^c^	Re-ranked^d^	Naïve	Re-ranked	Naïve	Re-ranked	Naïve	Re-ranked	Naïve	Re-ranked	Naïve	Re-ranked
0.95	2500	0.17	0.20	0.26	0.25	0.25	0.25	0.30	0.30	0.33	0.33	0.45	0.45
	5000	0.21	0.29	0.30	0.32	0.36	0.40	0.39	0.41	0.50	0.51	0.51	0.51
	7500	0.18	0.34	0.33	0.43	0.46	0.50	0.53	0.55	0.61	0.60	0.71	0.71
	10000	0.18	0.38	0.37	0.42	0.48	0.53	0.58	0.60	0.66	0.67	0.77	0.77

a See Table 2 for details of the simulation models.

b equal number of cases and controls.

c Naïve is standard ranking without correction for selection or genotyping error.

d Re-ranked is ranking by corrected statistic in Equation 1.

The simulation study also shows that the proposed re-ranking procedure can recover much of this lost power to identify the causal SNP, increasing the localization success rates by 1.5- to 3-fold in many cases (Table 3). When genotyping accuracy is high, the power lost due to tagging is small and so re-ranking tends to have little effect.

For studies using GWAS-based selection (scenario 1), the adverse effects of tagging and genotyping accuracy on localization success rate are strongest when the causal SNP is well tagged (larger *r*) and less accurately sequenced/imputed (smaller *ρ*) (Tables 3, 4 and S1). High-density GWAS followed up with low-coverage sequencing would fall into this category. Well-tagged causal SNPs tend to suffer from lower localization success rates because the perfectly genotyped tag often captures the association better than the imperfectly sequenced or imputed causal SNP. Re-ranking corrects this problem, so that the localization success rate does not depend on how well the causal SNP is tagged, except when the tag SNP is in fact the causal SNP. In this case, the tagging and genotyping accuracy effects actually increase the localization success rate. After re-ranking, the localization success rate is similar to levels seen when the tag is not causal. We consider this a minor tradeoff, because the causal SNP is unlikely to be found among the GWAS SNPs for a number of reasons: GWAS SNPs are typically selected independent of the phenotype of interest and post-GWAS SNPs tend to greatly outnumber GWAS SNPs.

When the discovery sample is also used for fine-mapping, but significance is not required at the GWAS-tag SNP (scenario 2), the genotyping accuracy effect alone could still considerably reduce power to identify the causal variant (Table 3). When an independent sample is used for fine-mapping (scenario 3, Table 3), localization success rates are very similar to those seen in scenario 2. In both cases, the re-ranking method improves the probability of correctly identifying the causal SNP. The improvement is most pronounced (2- to 4-fold improvement) when genotyping accuracy is low. When there is more than one causal variant (scenario 4, Table 3), we find that re-ranking effectively increases localization success rates for both causal SNPs. Imperfect call rates affect localization success rate in a similar manner to imperfect genotyping accuracy (scenario 5, Table 4). Equation (4) implies that a call rate 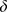 of 0.80 should affect the distribution of the causal SNP test statistic in the same manner as a sequencing accuracy *ρ* of 0.89, and this is borne out in our simulations. The re-ranking procedure corrects for both missing data and genotyping error to the same degree.

In some cases, investigators are more interested in delimiting a set of best candidate causal SNPs instead of a single top SNP. In the supplementary material, we include additional simulation results for this scenario. We define an alternative localization success rate metric as the probability that the causal SNP is in the top 10% of SNPs by rank (Table S2). Briefly, we examine the probability that the causal SNP is among the top 5 SNPs when there are 50 total SNPs (ranked by test statistic or re-ranking statistic). Without re-ranking, the probability that the causal SNP is in the top 10% of SNPs over the region is moderate. Re-ranking provides an improvement up to 1.8-fold.

### Application

Machiela *et al* [28] used the August 2010 release of the 1000 Genomes Project European-ancestry (EUR) panel to impute 11.6 million variants in 2,782 aggressive prostate cancer cases and 4,458 controls. These subjects were genotyped as part of the NCI Breast and Prostate Cancer (BPC3) Cohort Consortium aggressive prostate cancer GWAS [48], [49]; genotyping platforms varied across the seven BPC3 studies, although all used versions of the Illumina HumanHap arrays and most used the Illumina HumanHap 610 Quad array. The correlation between imputed genotype dosage and genotypes thus varied across studies. Imputation and association analyses using imputed genotype dosages were conducted separately for each study, and the association results were combined via fixed-effect meta-analysis. For each imputed SNP, studies with imputation r^2^<0.8 were excluded from the meta-analysis test statistic, leaving a total of 5.8 million GWAS and imputed SNPs.

Fine-mapping in the meta-analysis context ranks SNPs by the meta-analysis test statistic. Re-ranking requires that we compute the correlation between the meta-analysis test statistic on the Z-score scale (i.e. normally distributed test statistic) with and without accounting for genotyping error. Assume *Z_j_* is the normally distributed test statistic for study *j*, and *w_j_* is the weight for study *j*, the meta-analysis test statistic used for the standard naïve ranking is
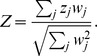
If 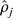 is an estimate of pair-wise correlation between the actual and imputed genotypes in study *j* (e.g. the square root of allelic-r^2^ [39], or ratio of variances r^2^ [24]), it follows that the estimated correlation between the meta-analysis test statistic computed with perfectly genotyped SNPs (*Z_act_*) and the meta-analysis test statistic computed with the observed imperfectly genotyped SNPs (*Z_obs_*) is
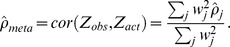
The re-ranking statistic in the meta-analysis case is
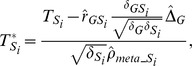
where 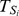 is the meta-analysis test statistic *Z* scaled for variance of 1.

**Figure 4:**
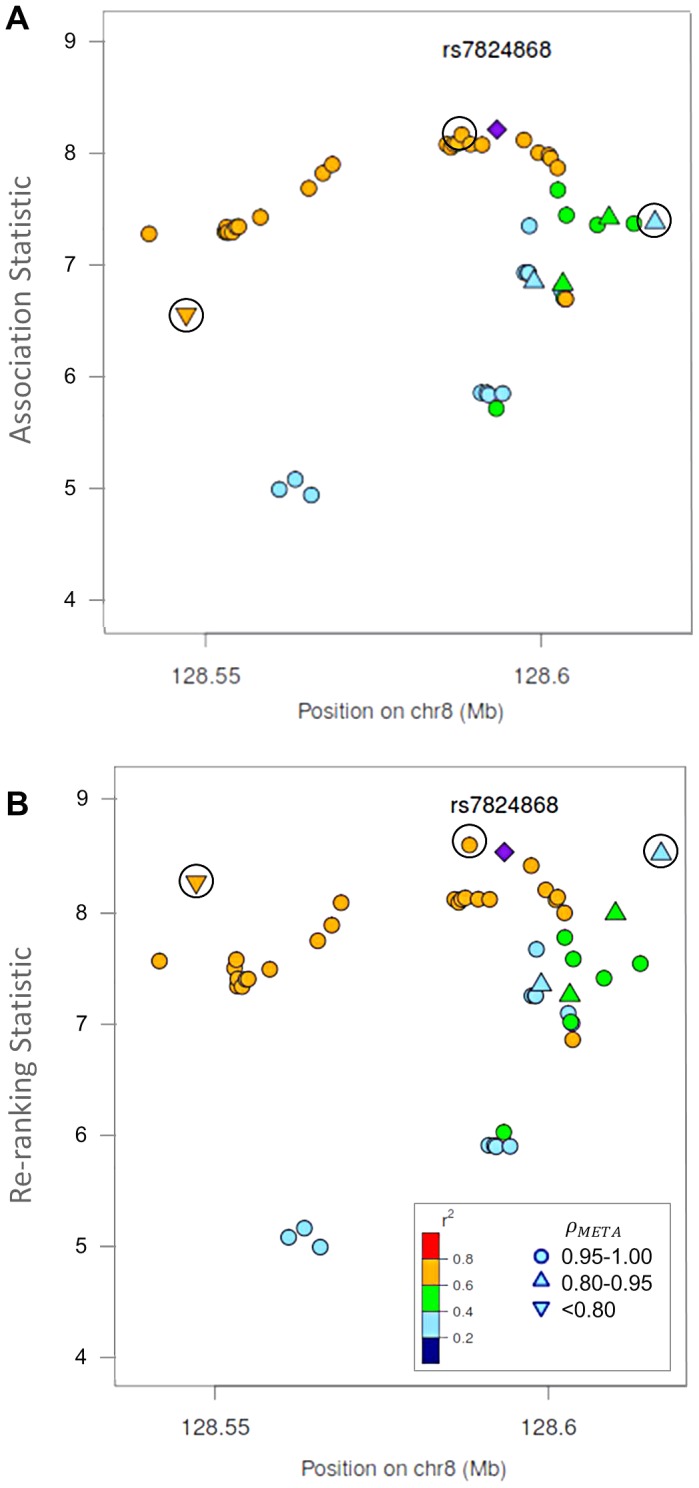
Naïve test statistics and re-ranking statistics for regions surrounding rs78246868 in the 8q24.21 region for association with prostate cancer risk. Naïve test statistics (**A**), and re-ranking statistics adjusting for genotyping accuracy (**B**) for SNPs in LD (r^2^>0.2) with rs78246868. Circles highlight SNPs whose rank changed considerably after re-ranking. Color indicates pair-wise correlation with the most significant SNP in the region selected based on the naïve ranking (purple diamond). Other shapes indicate genotyping accuracy over all 7 studies as measured by *ρ_meta_*. rs78246868 is no longer the most significant SNP in the region after re-ranking.

**Figure 5:**
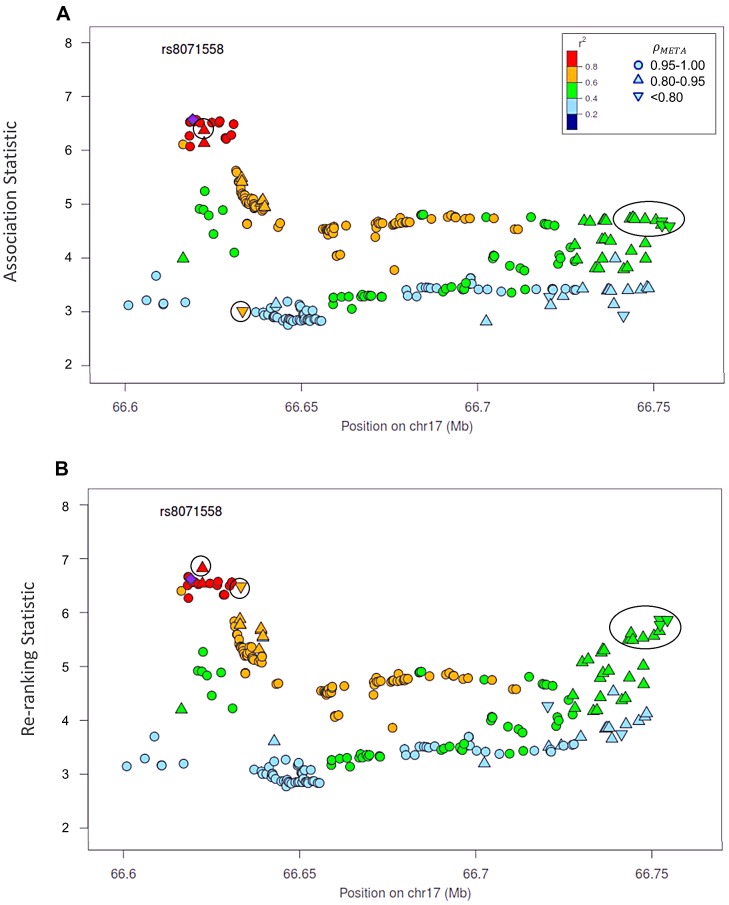
Naïve test statistics and re-ranking statistics for regions surrounding rs8071558 in the 17q24.3 region for association with prostate cancer risk. Naïve test statistics (**A**), and re-ranking statistics adjusting for genotyping accuracy (**B**) for SNPs in LD (r^2^>0.2) with rs8071558. Circles highlight SNPs whose rank changed considerably after re-ranking. Color indicates pair-wise correlation with the most significant SNP in the region selected based on the naïve ranking (purple diamond). Other shape indicates genotyping accuracy over all 7 studies as measured by *ρ_meta_*, rs8071558 is no longer the most significant SNP in the region after re-ranking.

Machiela *et al* [38] reported five statistically independent associated regions within the 8q24.21 locus and one for each of 11q13.3 and 17q24.3. We selected all SNPs in LD (r^2^>0.2) with the index SNP from each region for analyses (Figures 4 and 5, and Figures S11, S12, S13). In the application, we first ranked SNPs using the naïve test statistics [38]; and excluded any SNP with MAF <0.01; but unlike Machiela *et al* [38] we did not exclude any studies. Machiela *et al* selected significant regions by examining all imputed and genotyped SNPs at once and so we corrected for the imputation accuracy effect only (i.e. 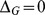).

Re-ranking identifies new top SNPs for 2 of the 3 associated loci: 8q24.21 and 17q24.3 (Figures 4 and 5 respectively). In addition to the most significant region at 8q24.21 (Figure 4), re-ranking also identifies a new top SNP for the third most significant region (Figure S11). For both regions re-ranking also identifies SNPs that may have otherwise been missed due to imperfect imputation. After re-ranking, 2 SNPs in the most significant region at the 8q24.21 locus (Figure 4) and 8 SNPs at the 17q24.3 locus (Figure 5) move from the lower ranks into the top 10 percent. On the other hand, SNPs in the top 10% are moved down by only a few ranks. In this way, re-ranking keeps highly significant SNPs identified by the naïve ranking and adds a few SNPs that would have otherwise been missed. When the top test statistics are of similar size, re-ranking may identify a new top SNP. When most SNPs are well-genotyped, re-ranking makes only subtle changes (Figure S11, S12 and S13).

There is one poorly imputed SNP at 17q24.3 (rs1014000, r^2^ = 0.20) that moves from the naïve rank of 245 to the new rank of 16 after adjustment. This SNP's apparent association is largely driven by data from a single study: the naïve rank in the EPIC study is 10. When we remove this study from the meta-analysis, the naïve rank is 306 and the adjusted rank is 119. No other SNP in the top 10% is this drastically affected when the EPIC study is removed from the analysis. In the meta-analysis context, we recommend examining top SNPs for heterogeneity among studies when re-ranking produces dramatically different results.

## Discussion

Overall, we observed that the tagging and genotyping accuracy effects are non-trivial sources of bias that could obscure association evidence at the causal SNP. The proposed re-ranking procedure is simple to implement and can substantially increase the probability of identifying the causal SNP. For low-coverage sequencing, we recommend the re-ranking method to improve causal SNP identification. For imputation and high-coverage sequencing, we recommend that unfiltered SNPs in associated regions be examined to see if correlation varies across SNPs and if so, we recommend adjustment with the re-ranking method. Large changes in rank should be carefully examined for underlying issues such as heterogeneity among meta-analysis studies or differential accuracy between cases and controls, and procedures to correct for these issues should be incorporated.

Re-ranking is most beneficial when genotyping accuracy is moderate to low, that is, the average correlation between the actual and estimated genotypes of post-GWAS (sequenced or imputed) SNPs is less than 0.97. A large number of post-GWAS SNPs in a study may appear to be significant, but when not all were directly genotyped with high accuracy, re-ranking can help select the most probable causal SNPs for follow-up. High density genotyping followed by low-coverage sequencing in the same sample can produce misleading results, as demonstrated by our simulations, so we do not recommend this design for identifying causal variants. Our re-ranking method tends to down-rank the tag SNP. If the tag SNP is suspected to be causal (e.g. based on prior study), we recommend examining the rank of the tag SNP using both the naïve and re-ranked methods when selecting SNPs for further study. Several imputation and sequencing software packages provide accurate estimates of *ρ* or quantities from which *ρ* can be computed [24], [39]. Re-ranking depends on accurate estimates of *ρ*. Recalibration of sequencing quality scores can greatly improve accuracy and so we recommend this step prior to re-ranking [27].

Re-ranking is especially important when study-specific factors exacerbate the effects of GWAS-based selection and genotyping error. Such factors include: high genetic diversity which makes sequencing reads difficult to align [27]; low LD among SNPs or lack of population-specific reference panels which makes some populations particularly difficult to impute (e.g. some African populations [50]); and imputation error which can be as high as 10% for these populations. Low MAF SNPs tend to suffer from both low power (which exacerbates the tagging effect) and high genotyping error. Re-ranking can be applied to rare and low MAF SNPs with allele counts large enough for test statistics to reach asymptotic normality. Very low (1×−2×) and extremely low (0.1×−0.5×) read depth sequencing has received recent attention as a way to maximize cost efficiency and make use of off-target sequencing data [29], [32]. Error rates for such regions would be both very high and highly variable among SNPs and so re-ranking to account for errors in the estimated genotypes would be crucial. When genotyping accuracy is extremely poor, the re-ranking method may not be able to sufficiently improve the localization success rate to ensure useful results. We recommend that investigators consider the accuracy thresholds recommended by the genotype calling or imputation algorithm they are using before re-ranking is applied.

We emphasize that re-ranking improves the localization success rate when applied to SNPs *under the alternative*, i.e. SNPs that are themselves causal or in LD with a causal SNP. Including null SNPs in the re-ranking procedure increases the number of SNPs the causal must out-compete, and so we recommend that only SNPs suspected to be under the alternative be included. In our application we included all SNPs that had squared pairwise correlation (*r^2^*) with the index SNP (most significant SNP in the region) greater than 0.2.

Existing methods that incorporate genotype uncertainty into tests for association to reduce power lost due to genotyping error or missing data [e.g. 51]–[54] do not completely recover lost power, and so the genotyping accuracy effect will remain. The simplest way to deal with genotype uncertainty in a test is to use the expected additive genotype (i.e. the posterior mean or dosage) in the standard linear or logistic regression. In this case, the re-ranking method can be applied using the allele dosages in place of called genotypes as described above. Guan and Stephens [55] compared several frequentist and Bayesian methods that incorporate genotype uncertainty into tests for association. The re-ranking procedure could be extended to any case where the correlation between test statistics or Bayes factors can be worked out.

We expect that re-ranking will play an important role as sequencing costs fall and GWAS platform coverage increases. Ultra-high density GWAS platforms are more likely to include tag SNPs in very high correlation with the causal SNP, which increases power to detect indirect association at the tag SNP. However, without re-ranking, strong tagging also decreases power to correctly identify the causal SNP in subsequent low-coverage sequencing. Advances in GWAS and sequencing platforms will allow researchers to drill down into lower MAFs and smaller effect sizes. Both low MAF and small effect size yield lower power, which exacerbates upward bias at the tag [20] and, therefore, the adverse tagging effect. Low MAF SNPs tend to suffer from higher error rates, which exacerbates the genotyping accuracy effect. Association study sample sizes will therefore need to continue to increase, so even as sequencing costs fall, it is anticipated that low-coverage will continue to be the most cost-effective design for many studies, despite the high genotyping error rates [27]. In conclusion, we anticipate that re-ranking to correct for the adverse effects of selection, tagging and differential genotyping accuracy rates among SNPs will continue to be important in candidate causal SNP identification for some time.

## Supporting Information

Figure S1 Distribution of SNP-specific read depth using the 1000 Genomes low-coverage pilot data on 351,456 SNPs from chromosome 1 in the CHB and JPT samples (October 2010 release; www.1000genomes.org/data).
(JPG)

Figure S2 Tagging effect decreases localization success rates with or without the selection effect, rare SNP. The expected values of the association test statistics at a tag SNP (red) and the causal SNP (black), shading from 25^th^–75^th^ percentiles (**A,C**), and the localization success rates (**B, D**) for association studies (1000 cases and 1000 controls) of one causal SNP (MAF = 0.02; OR = 1.5; perfect genotyping accuracy) and one tag SNP (MAF = 0.02; in varying degree of correlation with the causal SNP, *r = *0.2 to 1; perfect genotyping accuracy) with no selection for significance at the tag SNP (**A, B**) or selection at the tag SNP requiring the test statistic *T_G_* to be significant with p-value<0.05 (**C, D**).
(TIFF)

Figure S3 Tagging effect decreases localization success rates with or without the selection effect, high frequency SNP. The expected values of the association test statistics at a tag SNP (red) and the causal SNP (black), shading from 25^th^–75^th^ percentiles (**A,C**), and the localization success rates (**B, D**) for association studies (1000 cases and 1000 controls) of one causal SNP (MAF = 0.25; OR = 1.25; perfect genotyping accuracy) and one tag SNP (MAF = 0.25; in varying degree of correlation with the causal SNP, *r = *0.2 to 1; perfect genotyping accuracy) with no selection for significance at the tag SNP (**A, B**) or selection at the tag SNP requiring the test statistic *T_G_* to be significant with p-value<0.05 (**C, D**).
(TIF)

Figure S4 Genotyping accuracy effect further reduces localization success rates with or without the selection effect, rare SNP. Localization success rates for association studies (1000 cases and 1000 controls) of one causal SNP (MAF = 0.02; OR = 1.5; **imperfect genotyping accuracy** due to genotyping, sequencing or imputation errors resulting in correlation between the actual and estimated genotypes *ρ* ***_C_ =*** **0.80 (blue dash-dotted) to 1 (black solid)**) and one tag SNP (MAF = 0.02; in varying degree of correlation with the causal SNP, *r_CG_ = *0.2 to 1 (horizontal axis); perfect genotyping accuracy with *ρ* ***_G_ =*** **1**) with no selection for significance at the tag SNP (**A**) or selection at the tag SNP requiring the test statistic *T_G_* to be significant with p-value<0.05 (**B**).
(TIFF)

Figure S5 Genotyping accuracy effect further reduces localization success rates with or without the selection effect, high frequency SNP. Localization success rates for association studies (1000 cases and 1000 controls) of one causal SNP (MAF = 0.25; OR = 1.25; **imperfect genotyping accuracy** due to genotyping, sequencing or imputation errors resulting in correlation between the actual and estimated genotypes *ρ* ***_C_*** **= 0.80 (blue dash-dotted) to 1 (black solid)**) and one tag SNP (MAF = 0.25; in varying degree of correlation with the causal SNP, *r_CG_ = *0.2 to 1 (horizontal axis); perfect genotyping accuracy with *ρ* ***_G_ =*** **1**) with no selection for significance at the tag SNP (**A**) or selection at the tag SNP requiring the test statistic *T_G_* to be significant with p-value<0.05 (**B**).
(TIFF)

Figure S6 Figure well-tagged causal SNPs sequenced with low accuracy are unlikely to be correctly identified even as sample size increases, rare SNP. Localization success rates for association studies (50∶50 cases∶controls to 5000∶5000 cases∶controls, horizontal axis) of one causal SNP (MAF = 0.02; OR = 1.5; **imperfect genotyping accuracy** due to genotyping, sequencing or imputation errors resulting in correlation between the actual and estimated genotypes *ρ* ***_C_ =*** **0.95**) and one tag SNP (MAF = 0.02; **in high correlation with the causal SNP**, ***r_CG_ =*** **0.8 (purple solid) to 0.98 (red dashed)**; 100% genotyping accuracy with *ρ* ***_G_ =*** **1**) with no selection for significance at the tag SNP.
(TIFF)

Figure S7 Figure well-tagged causal SNPs sequenced with low accuracy are unlikely to be correctly identified even as sample size increases, high frequency SNP. Localization success rates for association studies (50∶50 cases∶controls to 5000∶5000 cases∶controls, horizontal axis) of one causal SNP (MAF = 0.25; OR = 1.25; **imperfect genotyping accuracy** due to genotyping, sequencing or imputation errors resulting in correlation between the actual and estimated genotypes *ρ* ***_C_ =*** **0.95**) and one tag SNP (MAF = 0.25; **in high correlation with the causal SNP**, ***r_CG_ =*** **0.8 (purple solid) to 0.98 (red dashed)**; 100% genotyping accuracy with *ρ* ***_G_ =*** **1**) with no selection for significance at the tag SNP.
(TIFF)

Figure S8 Tagging effect decreases localization success rates with or without the selection effect, 3 SNPs: 1 tag, 1 causal, 1 non-causal sequencing SNP. The expected values of the association test statistics at a tag SNP (red), the causal SNP (black), a non-causal sequencing SNP (green), shading from 25^th^–75^th^ percentiles (**A,C**), and the localization success rates (**B, D**) for association studies (1000 cases and 1000 controls) of one causal SNP (MAF = 0.02; correlation between causal and non-causal sequencing SNPs = 0.90, OR = 1.5; perfect genotyping accuracy) and one tag SNP (MAF = 0.02; in varying degree of correlation with the causal SNP, *r = *0.2 to 1; perfect genotyping accuracy) with no selection for significance at the tag SNP (**A, B**) or selection at the tag SNP requiring the test statistic *T_G_* to be significant with p-value<0.05 (**C, D**).
(TIFF)

Figure S9 Tagging effect decreases localization success rates with or without the selection effect 5 SNPs: 1 tag, 1 causal, 3 non-causal sequencing SNPs. The expected values of the association test statistics at a tag SNP (red), the causal SNP (black) and the maximum test statistic of the 3 non-causal sequencing SNPs (green), shading from 25^th^–75^th^ percentiles (**A,C**), and the localization success rates (**B, D**) for association studies (1000 cases and 1000 controls) of one causal SNP (MAF = 0.02; OR = 1.5; perfect genotyping accuracy) and one tag SNP (MAF = 0.02; correlation between causal and non-causal sequencing SNPs = 0.90, in varying degree of correlation with the causal SNP, *r = *0.2 to 1; perfect genotyping accuracy) with no selection for significance at the tag SNP (**A, B**) or selection at the tag SNP requiring the test statistic *T_G_* to be significant with p-value<0.05 (**C, D**).
(TIFF)

Figure S10 Tagging effect decreases localization success rates with or without the selection effect 7 SNPs: 1 tag, 1 causal, 5 non-causal sequencing SNPs. The expected values of the association test statistics at a tag SNP (red), the causal SNP (black) and the maximum test statistic of the 3 non-causal sequencing SNPs (green), shading from 25^th^–75^th^ percentiles (**A,C**), and the localization success rates (**B, D**) for association studies (1000 cases and 1000 controls) of one causal SNP (MAF = 0.02; OR = 1.5; perfect genotyping accuracy) and one tag SNP (MAF = 0.02; correlation between causal and non-causal sequencing SNPs = 0.90, in varying degree of correlation with the causal SNP, *r = *0.2 to 1; perfect genotyping accuracy) with no selection for significance at the tag SNP (**A, B**) or selection at the tag SNP requiring the test statistic *T_G_* to be significant with p-value<0.05 (**C, D**).
(TIFF)

Figure S11Naïve test statistics and re-ranking statistics for regions surrounding rs1016343 on 8q24.21 and rs34255287 on 11q13.3 for association with prostate cancer risk. Naïve test statistics (**A, B**), and re-ranking statistics adjusting for genotyping accuracy (**C, D**) for SNPs in LD (r^2^>0.2) with rs1016343 (**A, C**) or rs34255287 (**B, D**). Circles highlight SNPs whose rank changed considerably after re-ranking. Color indicates pair-wise correlation with the most significant SNP in the region. Shape indicates genotyping accuracy over all 7 cohorts as measured by 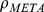, diamond is index SNP (most significant SNP from naive meta-analysis).(JPG)

Figure S12Naïve test statistics and re-ranking statistics for regions surrounding rs7816007 and rs6983267 on 8q24.21 for association with prostate cancer risk. Naïve test statistics (**A, B**), and re-ranking statistics adjusting for genotyping accuracy (**C, D**) for SNPs in LD (r^2^>0.2) with rs7816007 (**A, C**) or rs6983267 (**B, D**). Color indicates pair-wise correlation with the most significant SNP in the region. Shape indicates genotyping accuracy over all 7 cohorts as measured by 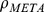, diamond is index SNP (most significant SNP from naive meta-analysis).(JPG)

Figure S13Naïve test statistics and re-ranking statistics for regions surrounding rs382434 on 8q24.21 for association with prostate cancer risk. Naïve test statistics (**A**), and re-ranking statistics adjusting for genotyping accuracy (**B**) for SNPs in LD (r^2^>0.2) with rs382434. Circles highlight SNPs whose rank changed considerably after re-ranking. Color indicates pair-wise correlation with the most significant SNP in the region. Shape indicates genotyping accuracy over all 7 cohorts as measured by 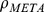, diamond is index SNP (most significant SNP from naive meta-analysis).(JPG)

Table S1 Trends in power and localization success rate due to tagging and genotyping accuracy effect.
(PDF)

Table S2 Alternative localization success rates^e^ for simulation Scenarios 2, 3, 4.
(PDF)

Text S1 Ranking SNPs to identify candidate causal SNPs.
(PDF)

Text S2 Derivation of distribution of GWAS and tag SNP test statistics.
(PDF)

Text S3 Low power exacerbates the selection effect.
(PDF)

Text S4 Tagging and coverage of GWAS platforms.
(PDF)
