# Supplementary figures and images for: Re-Ranking Sequencing Variants in the Post-GWAS Era for Accurate Causal Variant Identification
Source: PLoS Genet. 2013 Aug 8;9(8):e1003609. doi: 10.1371/journal.pgen.1003609 (PMC3738448; doi:10.1371/journal.pgen.1003609)

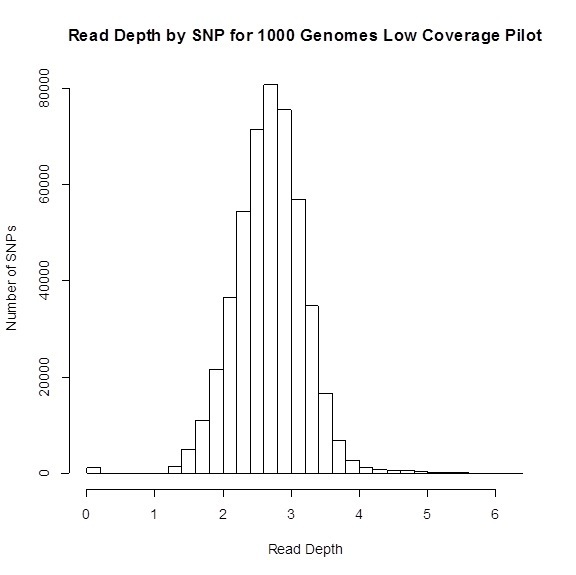

Supplement: Figure S1 — Distribution of SNP-specific read depth using the 1000 Genomes low-coverage pilot data on 351,456 SNPs from chromosome 1 in the CHB and JPT samples (October 2010 release; www.1000genomes.org/data). (JPG) [file pgen.1003609.s001.jpg]

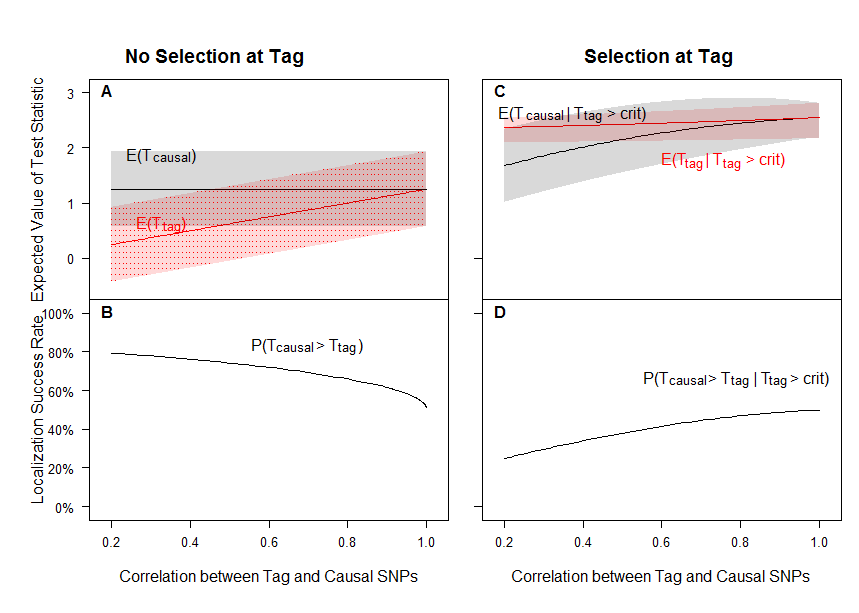

Supplement: Figure S2 — Tagging effect decreases localization success rates with or without the selection effect, rare SNP. The expected values of the association test statistics at a tag SNP (red) and the causal SNP (black), shading from 25th–75th percentiles (A,C), and the localization success rates (B, D) for association studies (1000 cases and 1000 controls) of one causal SNP (MAF = 0.02; OR = 1.5; perfect genotyping accuracy) and one tag SNP (MAF = 0.02; in varying degree of correlation with the causal SNP, r = 0.2 to 1; perfect genotyping accuracy) with no selection for significance at the tag SNP (A, B) or selection at the tag SNP requiring the test statistic TG to be significant with p-value<0.05 (C, D). (TIFF) [file pgen.1003609.s002.tiff]

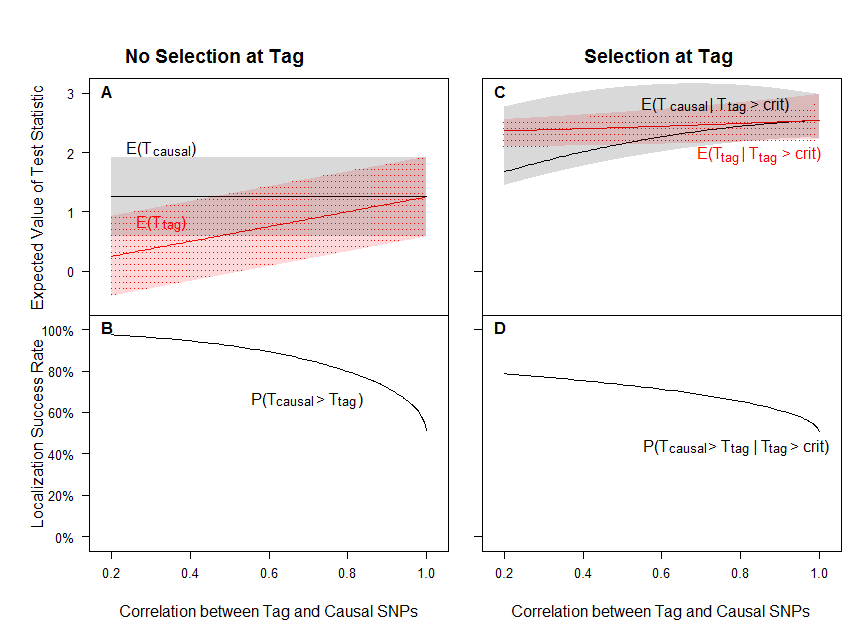

Supplement: Figure S3 — Tagging effect decreases localization success rates with or without the selection effect, high frequency SNP. The expected values of the association test statistics at a tag SNP (red) and the causal SNP (black), shading from 25th–75th percentiles (A,C), and the localization success rates (B, D) for association studies (1000 cases and 1000 controls) of one causal SNP (MAF = 0.25; OR = 1.25; perfect genotyping accuracy) and one tag SNP (MAF = 0.25; in varying degree of correlation with the causal SNP, r = 0.2 to 1; perfect genotyping accuracy) with no selection for significance at the tag SNP (A, B) or selection at the tag SNP requiring the test statistic TG to be significant with p-value<0.05 (C, D). (TIF) [file pgen.1003609.s003.tif]

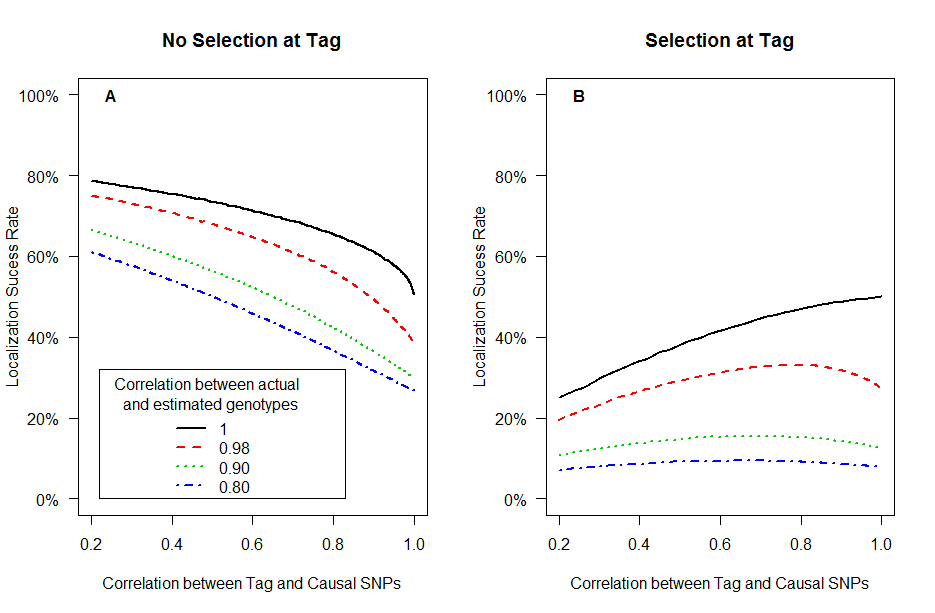

Supplement: Figure S4 — Genotyping accuracy effect further reduces localization success rates with or without the selection effect, rare SNP. Localization success rates for association studies (1000 cases and 1000 controls) of one causal SNP (MAF = 0.02; OR = 1.5; imperfect genotyping accuracy due to genotyping, sequencing or imputation errors resulting in correlation between the actual and estimated genotypes ρ C = 0.80 (blue dash-dotted) to 1 (black solid)) and one tag SNP (MAF = 0.02; in varying degree of correlation with the causal SNP, rCG = 0.2 to 1 (horizontal axis); perfect genotyping accuracy with ρ G = 1) with no selection for significance at the tag SNP (A) or selection at the tag SNP requiring the test statistic TG to be significant with p-value<0.05 (B). (TIFF) [file pgen.1003609.s004.tiff]

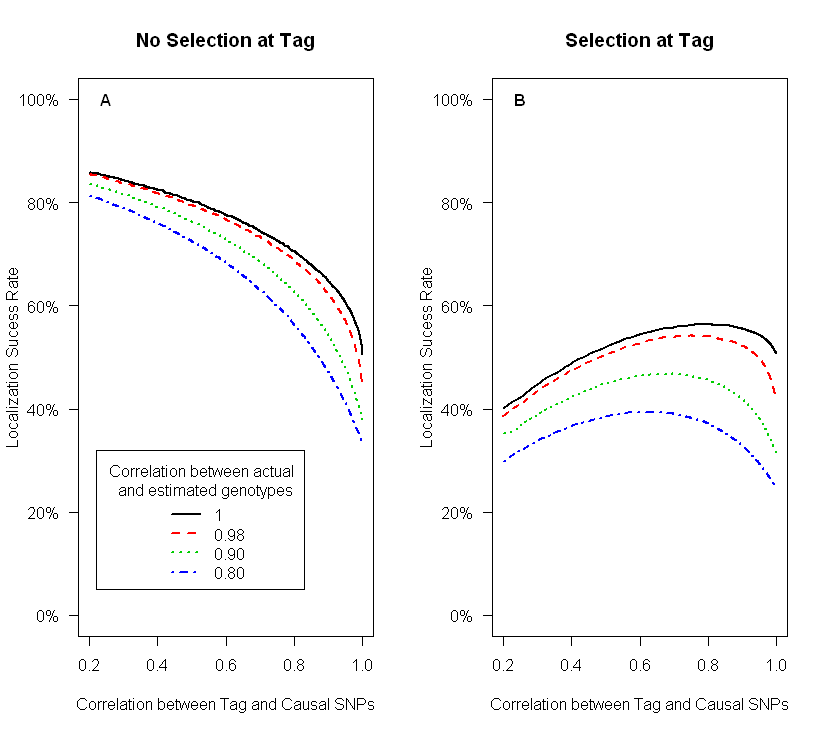

Supplement: Figure S5 — Genotyping accuracy effect further reduces localization success rates with or without the selection effect, high frequency SNP. Localization success rates for association studies (1000 cases and 1000 controls) of one causal SNP (MAF = 0.25; OR = 1.25; imperfect genotyping accuracy due to genotyping, sequencing or imputation errors resulting in correlation between the actual and estimated genotypes ρ C = 0.80 (blue dash-dotted) to 1 (black solid)) and one tag SNP (MAF = 0.25; in varying degree of correlation with the causal SNP, rCG = 0.2 to 1 (horizontal axis); perfect genotyping accuracy with ρ G = 1) with no selection for significance at the tag SNP (A) or selection at the tag SNP requiring the test statistic TG to be significant with p-value<0.05 (B). (TIFF) [file pgen.1003609.s005.tiff]

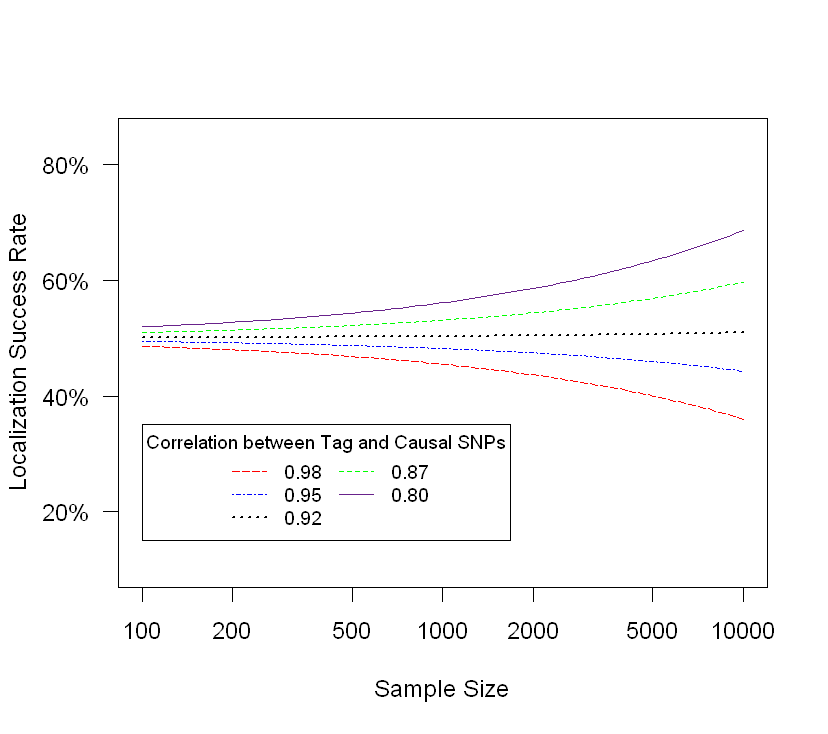

Supplement: Figure S6 — Figure well-tagged causal SNPs sequenced with low accuracy are unlikely to be correctly identified even as sample size increases, rare SNP. Localization success rates for association studies (50∶50 cases∶controls to 5000∶5000 cases∶controls, horizontal axis) of one causal SNP (MAF = 0.02; OR = 1.5; imperfect genotyping accuracy due to genotyping, sequencing or imputation errors resulting in correlation between the actual and estimated genotypes ρ C = 0.95) and one tag SNP (MAF = 0.02; in high correlation with the causal SNP, rCG = 0.8 (purple solid) to 0.98 (red dashed); 100% genotyping accuracy with ρ G = 1) with no selection for significance at the tag SNP. (TIFF) [file pgen.1003609.s006.tiff]

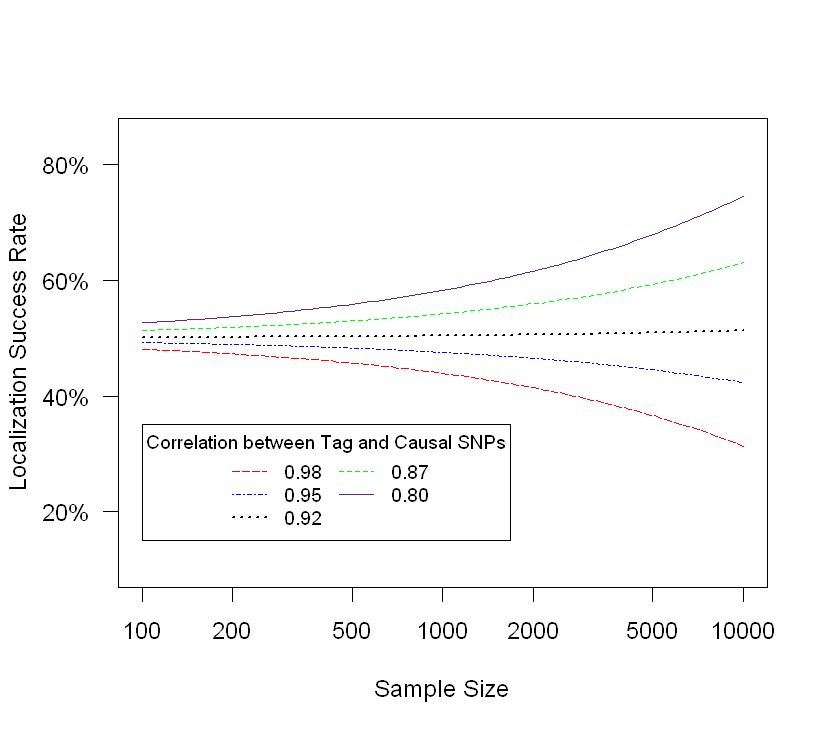

Supplement: Figure S7 — Figure well-tagged causal SNPs sequenced with low accuracy are unlikely to be correctly identified even as sample size increases, high frequency SNP. Localization success rates for association studies (50∶50 cases∶controls to 5000∶5000 cases∶controls, horizontal axis) of one causal SNP (MAF = 0.25; OR = 1.25; imperfect genotyping accuracy due to genotyping, sequencing or imputation errors resulting in correlation between the actual and estimated genotypes ρ C = 0.95) and one tag SNP (MAF = 0.25; in high correlation with the causal SNP, rCG = 0.8 (purple solid) to 0.98 (red dashed); 100% genotyping accuracy with ρ G = 1) with no selection for significance at the tag SNP. (TIFF) [file pgen.1003609.s007.tiff]

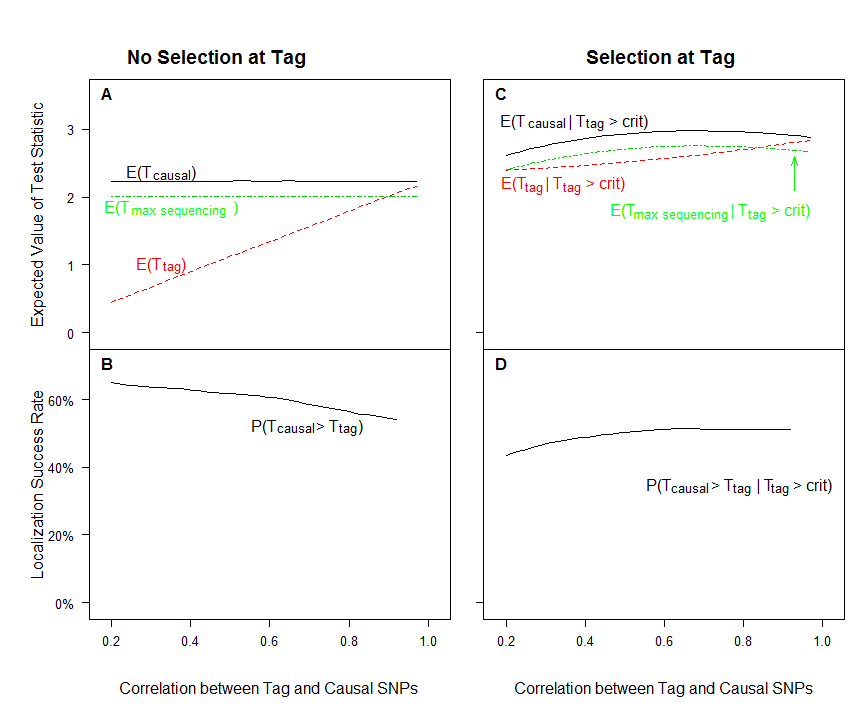

Supplement: Figure S8 — Tagging effect decreases localization success rates with or without the selection effect, 3 SNPs: 1 tag, 1 causal, 1 non-causal sequencing SNP. The expected values of the association test statistics at a tag SNP (red), the causal SNP (black), a non-causal sequencing SNP (green), shading from 25th–75th percentiles (A,C), and the localization success rates (B, D) for association studies (1000 cases and 1000 controls) of one causal SNP (MAF = 0.02; correlation between causal and non-causal sequencing SNPs = 0.90, OR = 1.5; perfect genotyping accuracy) and one tag SNP (MAF = 0.02; in varying degree of correlation with the causal SNP, r = 0.2 to 1; perfect genotyping accuracy) with no selection for significance at the tag SNP (A, B) or selection at the tag SNP requiring the test statistic TG to be significant with p-value<0.05 (C, D). (TIFF) [file pgen.1003609.s008.tiff]

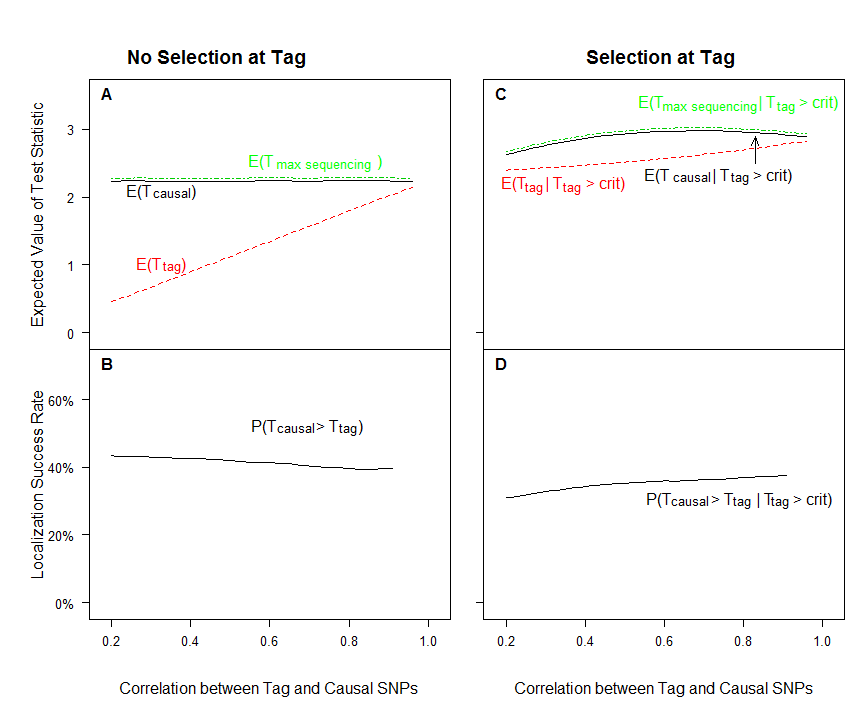

Supplement: Figure S9 — Tagging effect decreases localization success rates with or without the selection effect 5 SNPs: 1 tag, 1 causal, 3 non-causal sequencing SNPs. The expected values of the association test statistics at a tag SNP (red), the causal SNP (black) and the maximum test statistic of the 3 non-causal sequencing SNPs (green), shading from 25th–75th percentiles (A,C), and the localization success rates (B, D) for association studies (1000 cases and 1000 controls) of one causal SNP (MAF = 0.02; OR = 1.5; perfect genotyping accuracy) and one tag SNP (MAF = 0.02; correlation between causal and non-causal sequencing SNPs = 0.90, in varying degree of correlation with the causal SNP, r = 0.2 to 1; perfect genotyping accuracy) with no selection for significance at the tag SNP (A, B) or selection at the tag SNP requiring the test statistic TG to be significant with p-value<0.05 (C, D). (TIFF) [file pgen.1003609.s009.tiff]

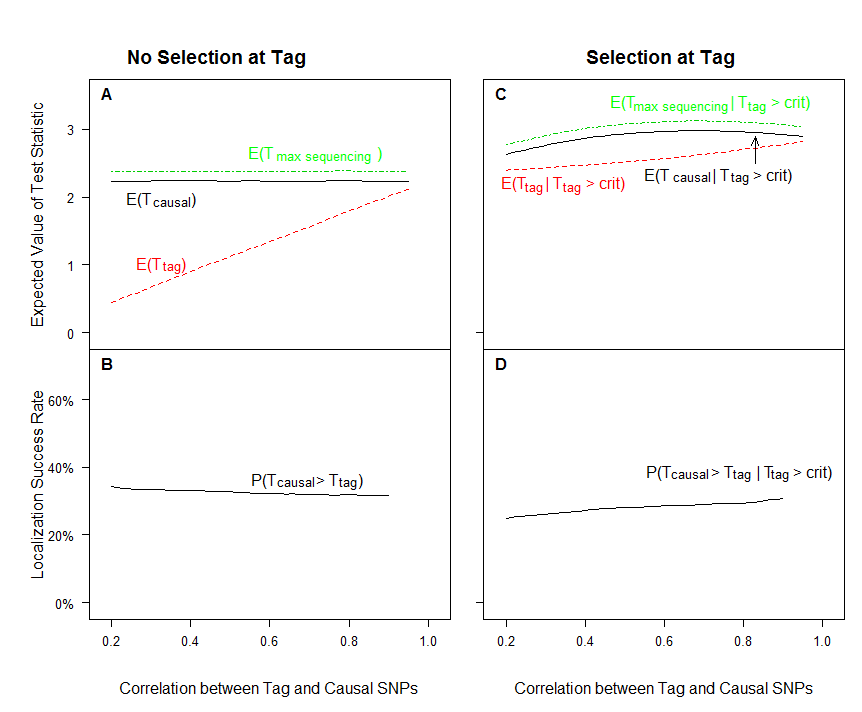

Supplement: Figure S10 — Tagging effect decreases localization success rates with or without the selection effect 7 SNPs: 1 tag, 1 causal, 5 non-causal sequencing SNPs. The expected values of the association test statistics at a tag SNP (red), the causal SNP (black) and the maximum test statistic of the 3 non-causal sequencing SNPs (green), shading from 25th–75th percentiles (A,C), and the localization success rates (B, D) for association studies (1000 cases and 1000 controls) of one causal SNP (MAF = 0.02; OR = 1.5; perfect genotyping accuracy) and one tag SNP (MAF = 0.02; correlation between causal and non-causal sequencing SNPs = 0.90, in varying degree of correlation with the causal SNP, r = 0.2 to 1; perfect genotyping accuracy) with no selection for significance at the tag SNP (A, B) or selection at the tag SNP requiring the test statistic TG to be significant with p-value<0.05 (C, D). (TIFF) [file pgen.1003609.s010.tiff]

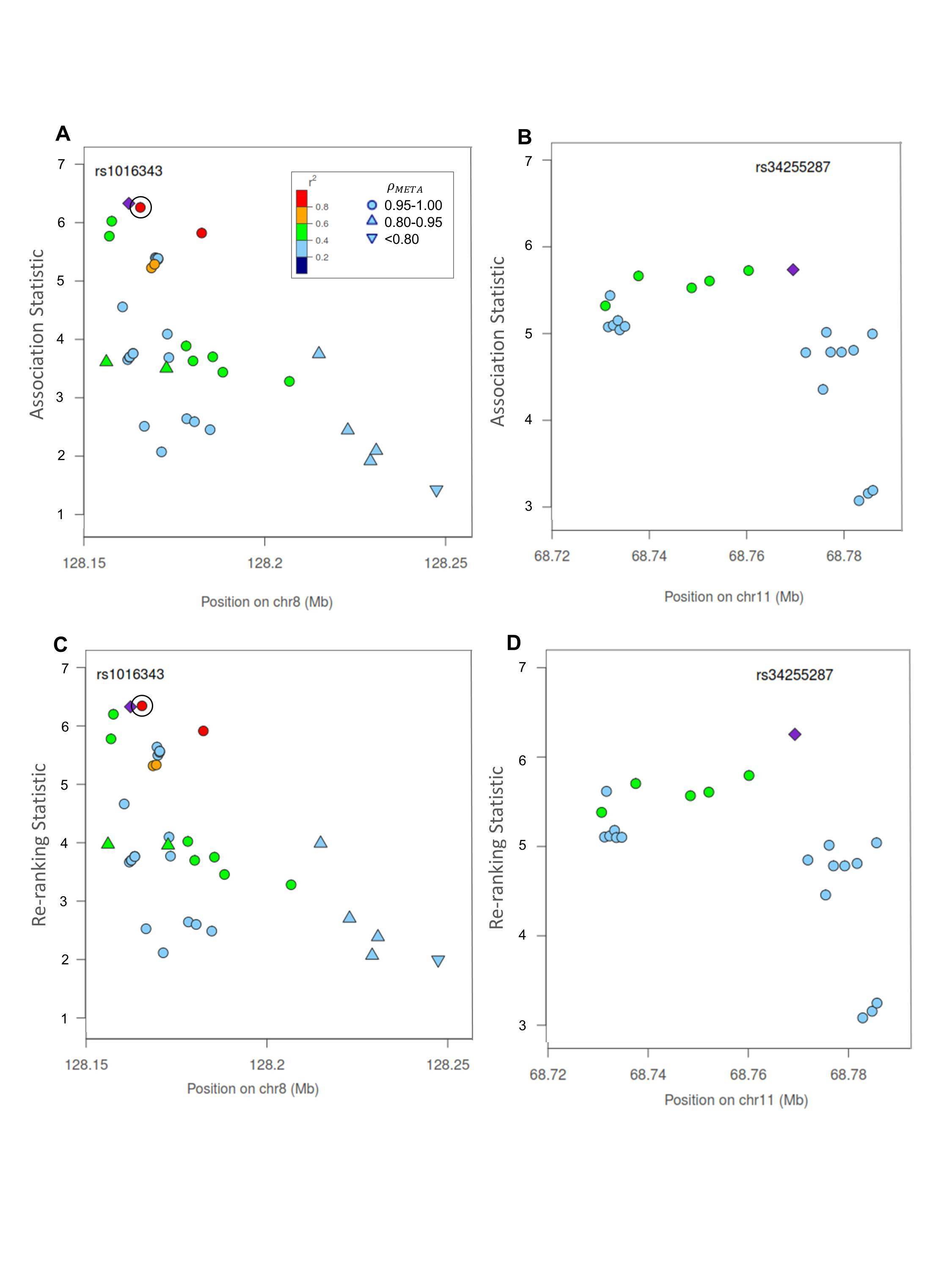

Supplement: Figure S11 — Naïve test statistics and re-ranking statistics for regions surrounding rs1016343 on 8q24.21 and rs34255287 on 11q13.3 for association with prostate cancer risk. Naïve test statistics (A, B), and re-ranking statistics adjusting for genotyping accuracy (C, D) for SNPs in LD (r2>0.2) with rs1016343 (A, C) or rs34255287 (B, D). Circles highlight SNPs whose rank changed considerably after re-ranking. Color indicates pair-wise correlation with the most significant SNP in the region. Shape indicates genotyping accuracy over all 7 cohorts as measured by , diamond is index SNP (most significant SNP from naive meta-analysis). (JPG) [file pgen.1003609.s011.jpg]

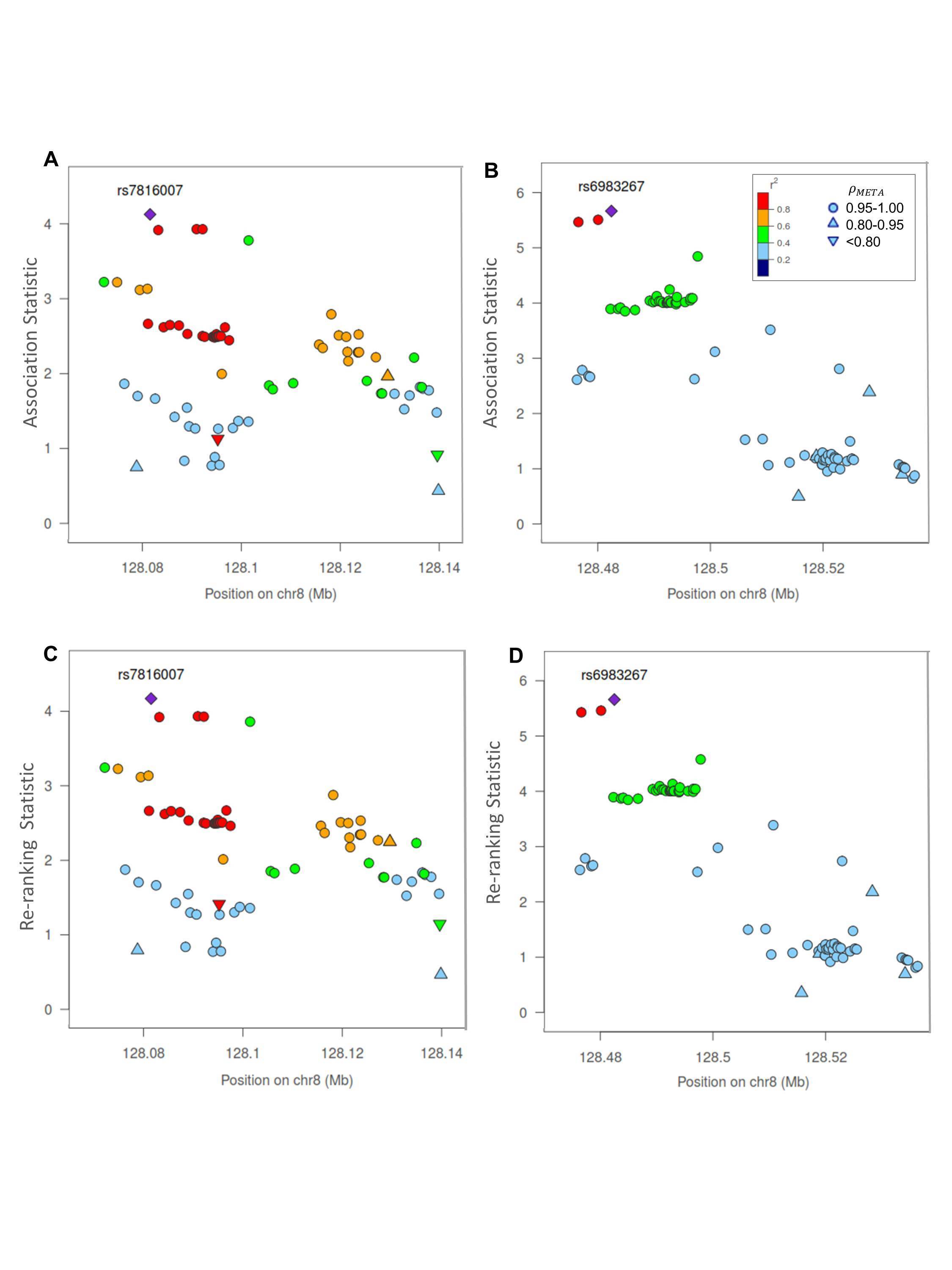

Supplement: Figure S12 — Naïve test statistics and re-ranking statistics for regions surrounding rs7816007 and rs6983267 on 8q24.21 for association with prostate cancer risk. Naïve test statistics (A, B), and re-ranking statistics adjusting for genotyping accuracy (C, D) for SNPs in LD (r2>0.2) with rs7816007 (A, C) or rs6983267 (B, D). Color indicates pair-wise correlation with the most significant SNP in the region. Shape indicates genotyping accuracy over all 7 cohorts as measured by , diamond is index SNP (most significant SNP from naive meta-analysis). (JPG) [file pgen.1003609.s012.jpg]

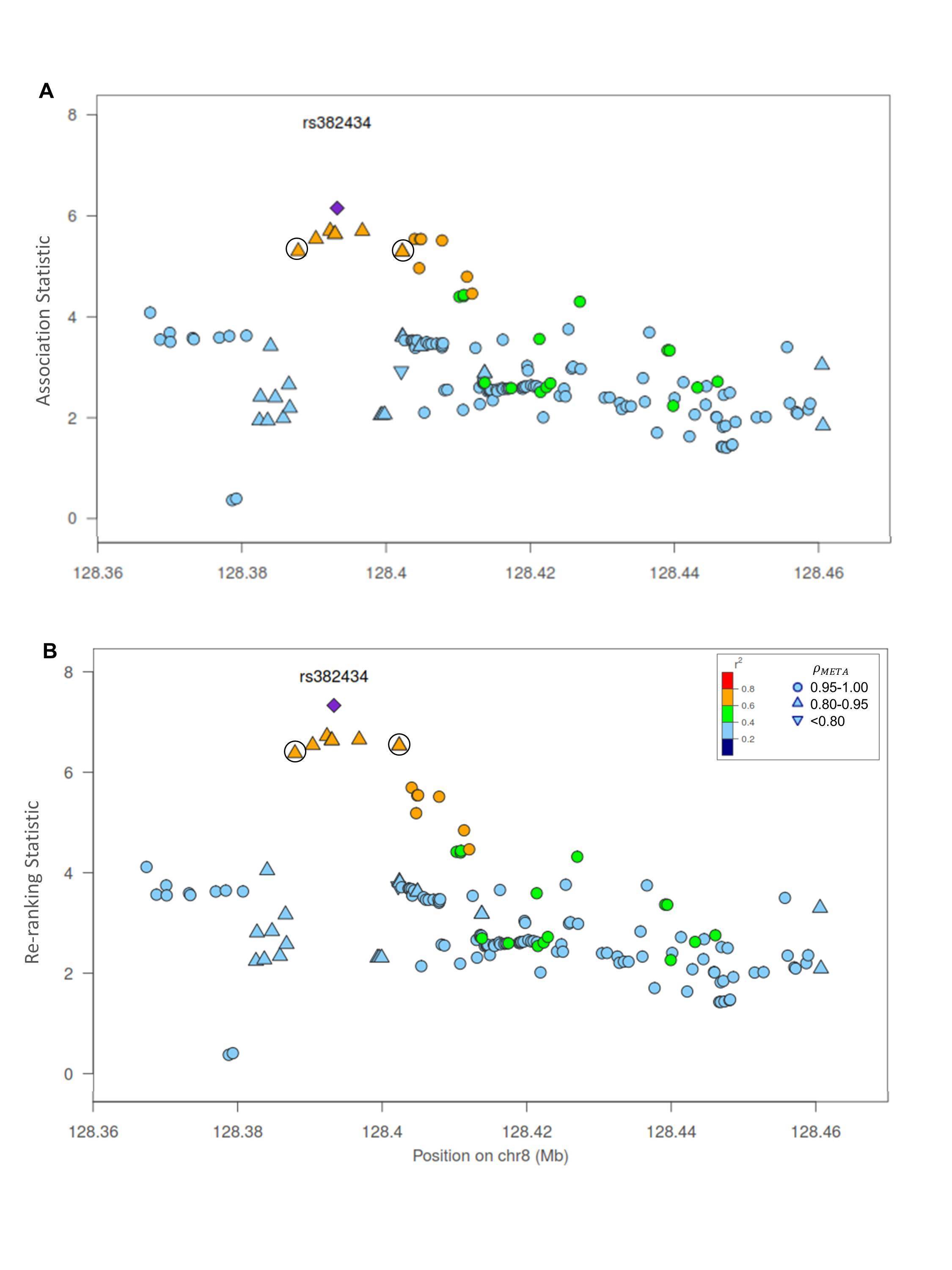

Supplement: Figure S13 — Naïve test statistics and re-ranking statistics for regions surrounding rs382434 on 8q24.21 for association with prostate cancer risk. Naïve test statistics (A), and re-ranking statistics adjusting for genotyping accuracy (B) for SNPs in LD (r2>0.2) with rs382434. Circles highlight SNPs whose rank changed considerably after re-ranking. Color indicates pair-wise correlation with the most significant SNP in the region. Shape indicates genotyping accuracy over all 7 cohorts as measured by , diamond is index SNP (most significant SNP from naive meta-analysis). (JPG) [file pgen.1003609.s013.jpg]
