# Supplementary material for: Re-Ranking Sequencing Variants in the Post-GWAS Era for Accurate Causal Variant Identification
Source: PLoS Genet. 2013 Aug 8;9(8):e1003609. doi: 10.1371/journal.pgen.1003609 (PMC3738448; doi:10.1371/journal.pgen.1003609)
Supplement: Table S1 — Trends in power and localization success rate due to tagging and genotyping accuracy effect. (PDF) [file pgen.1003609.s014.pdf]

Table S1. Trends in power and localization success rate due to tagging and genotyping accuracy effect

	Sequence Independent Sample, Genotyping 100% Accurate[a]		Sequence Independent Sample, Genotyping 97% Accurate[b]		Sequence GWAS Discovery Sample, Genotyping 97% Accurate[c]	
Tagging r[d]	Power to Detect[e]	Localization Success Rate[f]	Power to Detect	Localization Success Rate	Power to Detect	Localization Success Rate
0.78	0.24	0.61	0.04	0.36	0.50	0.22
0.85	0.19	0.45	0.09	0.25	0.52	0.15
0.93	0.24	0.40	0.10	0.17	0.42	0.09
0.98	0.23	0.36	0.08	0.08	0.23	0.04

Simulation parameters as describe in Table 2, Simulation 1 except as noted below
[a] All SNPs genotyped with perfect accuracy, regions selected by prior information
[b] GWAS SNPs genotyped perfectly, sequencing SNPs genotyped imperfectly (correlation between called and actual genotypes ρ=0.86), regions selected by prior information
[c] GWAS SNPs genotyped perfectly, sequencing SNPs genotyped imperfectly (correlation between called and actual genotypes ρ=0.86), regions selected by significant (p<5e-7) GWAS tag SNP
[d] Correlation between the tag and causal SNPs
[e] Probability causal SNP significant (p<5e-7)
[f] Probability causal SNP is most significant in region

In Table S1, we simulated data for 3 cases:

(1) **Sequence Independent Sample, Genotyping 100% Accurate.** We simulated GWAS and sequencing data as described in Scenario 1 of the main text except that genotyping accuracy was 100% and fine-mapping was not conditional on genome-wide significance.

(2) **Sequence Independent Sample, Genotyping 97% Accurate**. We simulate data as in (1) except that genotyping accuracy is 97%.

(3) **Sequence GWAS Discovery Sample, Genotyping 97% Accurate** We simulate data as in (2) except that fine-mapping is conditional on genome-wide significance at the tag SNP

The results show that when fine-mapping is performed in an independent sample with 100% accurate genotyping, the power to detect the original effect at the causal SNP as well as the

localization success rate is high. When genotyping error is introduced, both power and localization success rate fall considerably. Re-using the original successful GWAS for the fine-mapping stage increases the probability that the causal SNP will be genome-wide significant because it is conditional on genome-wide significance at the GWAS tag SNP. However, re-using the GWAS data considerably decreases the probability that the causal SNP will be top-ranked.
