# Supplementary material for: Re-Ranking Sequencing Variants in the Post-GWAS Era for Accurate Causal Variant Identification
Source: PLoS Genet. 2013 Aug 8;9(8):e1003609. doi: 10.1371/journal.pgen.1003609 (PMC3738448; doi:10.1371/journal.pgen.1003609)
Supplement: Table S2 — Alternative localization success ratese for simulation Scenarios 2, 3, 4. (PDF) [file pgen.1003609.s015.pdf]

Table S2. Alternative Localization success rates[e] for simulation Scenarios 2, 3, 4

Correlation between the tag and causal SNPs, r	Sample size[b]	Average correlation between the actual and estimated genotypes of sequencing SNPs, ρ_{si}											
		◄ Low-coverage Sequencing ►								High-coverage Sequencing			
		0.82		0.86		0.90		0.95		0.97		1.00	
		Naïve[c]	Re-ranked[d]	Naïve	Re-ranked	Naïve	Re-ranked	Naïve	Re-ranked	Naïve	Re-ranked	Naïve	Re-ranked
		**Scenario 2[a]**											
0.95	2500	0.57	0.72	0.57	0.74	0.66	0.79	0.74	0.82	0.77	0.81	0.87	0.87
	5000	0.55	0.85	0.63	0.89	0.75	0.90	0.87	0.95	0.92	0.94	0.96	0.96
	7500	0.58	0.92	0.65	0.94	0.78	0.94	0.90	0.97	0.96	0.98	0.98	0.98
	10000	0.60	0.94	0.66	0.96	0.78	0.98	0.92	0.99	0.97	0.99	1.00	1.00
		**Scenario 3[a]**											
0.95	2500	0.52	0.70	0.59	0.72	0.63	0.74	0.76	0.80	0.79	0.82	0.86	0.86
	5000	0.59	0.84	0.62	0.88	0.75	0.90	0.87	0.92	0.93	0.95	0.96	0.96
	7500	0.59	0.93	0.67	0.95	0.77	0.96	0.90	0.97	0.96	0.98	0.99	0.99
	10000	0.60	0.95	0.70	0.98	0.77	0.98	0.92	0.99	0.98	1.00	1.00	1.00
		**Scenario 4[a]**											
0.8	2500	0.27	0.42	0.31	0.43	0.33	0.42	0.38	0.45	0.43	0.45	0.46	0.46
	5000	0.30	0.43	0.33	0.46	0.34	0.47	0.41	0.45	0.45	0.48	0.51	0.51
	7500	0.28	0.50	0.32	0.49	0.36	0.50	0.41	0.52	0.47	0.51	0.52	0.52
	10000	0.29	0.50	0.32	0.45	0.34	0.51	0.41	0.50	0.46	0.54	0.54	0.54
0.93	2500	0.29	0.41	0.32	0.40	0.33	0.40	0.37	0.42	0.40	0.41	0.45	0.45
	5000	0.30	0.44	0.32	0.44	0.33	0.45	0.37	0.47	0.43	0.46	0.48	0.48
	7500	0.30	0.51	0.34	0.53	0.36	0.53	0.42	0.55	0.50	0.55	0.52	0.52
	10000	0.29	0.48	0.32	0.53	0.36	0.51	0.39	0.50	0.43	0.52	0.52	0.52

[a] See Table 2 for details of the simulation models; scenario 4 has two causal loci
[b] equal number of cases and controls for Scenario 2,3,4
[c] Naïve is standard ranking without correction for selection or genotyping error
[d] Re-ranked is ranking by corrected statistic in Equation 1
[e] Localization success rate defined as probability causal SNP is in top 10% of SNPs ranked by test statistic, re-ranking statistic

Simulation Details

In some cases, investigators might be interested in selecting a set of candidate SNPs instead of a single top one. Here we define an alternative localization rate metric as the probability that the causal SNP is among the top 10% of the SNPs by rank. For this table, we simulated data in the same manner as the described in the main text for Scenarios 2-4, except that there are a total of 50 SNPs in the region: 1 tag, 1 causal and 48 non-causal sequencing SNPs.
