# Supplementary material for: Re-Ranking Sequencing Variants in the Post-GWAS Era for Accurate Causal Variant Identification
Source: PLoS Genet. 2013 Aug 8;9(8):e1003609. doi: 10.1371/journal.pgen.1003609 (PMC3738448; doi:10.1371/journal.pgen.1003609)
Supplement: Text S1 — Ranking SNPs to identify candidate causal SNPs. (PDF) [file pgen.1003609.s016.pdf]

Text S1: Ranking SNPs to Identify Candidate Causal SNPs

Ranking of SNPs over an associated region is commonly used in GWAS and imputation studies and we expect it to be used in sequencing studies. GWAS and imputation studies typically report the top-ranked SNP for each associated locus. For example, the primary publication for the WTCCC 1 study reports the single most significant imputed or directly genotyped SNP (i.e. the top ranked SNP) across each associated region.

Follow-up studies usually attempt replication for the top SNPs in each region, not the top SNPs over the entire genome. Selecting top SNPs over the genome would favor the most strongly associated loci and leave out associated regions with weaker signals. While maximizing SNPs over the genome would result in the highest proportion of SNPs being replicated, it is regions of marginal significance that actually require further evidence. Selecting the most promising SNP from each region gives follow up studies the best chance to verify all truly associated loci.

Not all loci require follow up, some loci will have already been fully explored by previous studies. Prioritization of SNPs at the locus level instead of the genome level allows researchers to select the loci that actually require follow up. For example, Todd et al [54] followed up the WTCCC T1D novel associations but not the loci that had previously been confirmed.

Next generation sequencing is a relatively new technique whose full potential will unfold over the coming years. NGS provides data similar to dense genotyping arrays, though at a more comprehensive level and so analysis will likely be similar. For dense array association studies, researchers typically report the top SNP in each associated region. For example, Liu et al [55] report the top-ranked SNP at each significant locus in UK-PBC Consortium's primary biliary

cirrhosis dense genotyping array association study. Given the cost of functional studies, only a few top candidate SNPs will be moved forward and so narrowing candidates down to a handful of top-ranked SNPs will be critical to success.

Additional References for Text S2

54. Todd JA, Walker NM, Cooper JD, Smyth DJ, Downes K et. al. (2007) Robust associations of four new chromosome regions from genome-wide analyses of type 1 diabetes. Nat. Genet. 39: 857-864.

55. Liu JZ, Mohamed AA, Gaffney DJ, Mells GF, Hostins L et.al. (2012) Dense fine-mapping study identifies new susceptibility loci for primary biliary cirrhosis. Nat. Genet. 44:1137-1141
