# Supplementary material for: Re-Ranking Sequencing Variants in the Post-GWAS Era for Accurate Causal Variant Identification
Source: PLoS Genet. 2013 Aug 8;9(8):e1003609. doi: 10.1371/journal.pgen.1003609 (PMC3738448; doi:10.1371/journal.pgen.1003609)
Supplement: Text S2 — Derivation of distribution of GWAS and tag SNP test statistics. (PDF) [file pgen.1003609.s017.pdf]

Text S2. Derivation of Distribution of GWAS and Tag SNP Test Statistics

Let $S_1 \ldots S_k$ be the sequencing SNPs in the disease-associated region, one of which is the causal SNP, denoted as *C*. The GWAS tag SNP is denoted as *G*. Let T_G be the test statistic at the tag SNP, and $T_{S_1}, T_{S_2}, \ldots, T_{S_k}$ be the vector of test statistics for the sequencing SNPs. We define the pairwise correlations as follows: r_{CS_i} is the pairwise correlation between the causal SNP and sequencing SNP *i*, r_{GS_i} is the pairwise correlation between the GWAS tag SNP and sequencing SNP *i*, $r_{S_iS_j}$ is the pairwise correlation between sequencing SNPs *i* and *j*, r_{CG} is the pairwise correlation between the causal SNP and the GWAS tag SNP ($r_{CG} > 0$ without loss of generality). One of the sequencing SNPs is causal, so for one of $i=1 \ldots k$, $T_{S_i} = T_C$, $r_{CS_i} = 1$ and $r_{GS_i} = r_{CG}$.

The multivariate normal (MVN) joint distribution of the test statistics at the tag and sequencing SNPs is

$$\begin{pmatrix} T_G \\ T_{S_1} \\ T_{S_2} \\ \vdots \\ T_{S_k} \end{pmatrix} \sim N\left(\sqrt{n}\mu_C \begin{pmatrix} r_{CG} \\ r_{CS_1} \\ r_{CS_2} \\ \vdots \\ r_{CS_k} \end{pmatrix}, \begin{pmatrix} 1 & r_{GS_1} & r_{GS_2} & \cdots & r_{GS_k} \\ r_{GS_1} & 1 & r_{S_1S_2} & \cdots & r_{S_1S_k} \\ r_{GS_2} & r_{S_1S_2} & 1 & \cdots & r_{S_2S_k} \\ \vdots & \vdots & \vdots & \ddots & \vdots \\ r_{GS_k} & r_{S_1S_k} & r_{S_2S_k} & \cdots & 1 \end{pmatrix} \right). \qquad \text{Eqn A}$$

Note that although the GWAS tag SNP is first in the vector, the physical location of the tag SNP is likely to be in the middle of the sequencing SNPs. In Equation A, we assume no genotyping error and no prior selection of the region for a significant tag SNP association.

The expected value of the test statistic at a particular SNP is proportional to its correlation with the causal SNP. The expected value is largest when correlation with the causal SNP is 1, i.e. the SNP is causal or in perfect LD with the causal SNP. Further details for the derivation of Eqn A

are below.

Tagging Effect

Selection of significant regions via GWAS tag SNPs induces upward bias in the magnitude of observed tag SNP associations. When GWAS samples are sequenced, the bias at the tag carries through to sequencing SNPs in high LD with the tag. This effect attenuates as correlation between the tag and sequencing SNPs breaks down [20].

Conditional on the value of the observed test statistic $T_{G_{obs}}$ at the tag SNP, the joint distribution of the test statistics at the sequencing SNPs is

$$\left\{ \begin{pmatrix} T_{S_1} \\ T_{S_2} \\ \vdots \\ T_{S_k} \end{pmatrix} \middle| T_G = T_{G_{obs}} \right\} \sim N\left(\sqrt{n}\mu_C \begin{pmatrix} r_{CS_1} \\ r_{CS_2} \\ \vdots \\ \underline{r_{CS_K}} \end{pmatrix} + \begin{pmatrix} r_{GS_1} \\ r_{GS_2} \\ \vdots \\ r_{GS_K} \end{pmatrix} (T_{G_{obs}} - r_{GC}\mu_C\sqrt{n}), \Sigma \right) \quad , \qquad \text{Eqn B}$$

where the elements of Σ are $\Sigma_{ij} = r_{S_iS_j} - r_{GS_i} r_{GS_j} \quad i = 1...k, j = 1...k$.

We assume, without loss of generality that the tag SNP genotype is coded so that $T_{G_{obs}} > 0$. If we select for a large tag SNP test statistic, then the expected value of $T_{G_{obs}} - r_{GC}\mu_C\sqrt{n} > 0$. We call $T_{G_{obs}} - r_{GC}\mu_C\sqrt{n}$ the tagging bias term. The tagging bias term carries through to the sequencing SNPs in proportion to the correlation between the tag and sequencing SNPs, r_{GS_K}. The combination of distortion at the tag and attenuation due to LD distorts the signal (i.e. the expected value of the test statistic at each SNP) so that SNPs in high LD with the tag are more likely to be top-ranked, even when they are non-causal. We call this phenomenon the tagging effect. The re-ranking procedure first estimates the effect of selection on the tag SNP test

statistic $\left(T_{\mathrm{G_{obs}}} - T_{\mathrm{G_{BOOT}}}\right)$, then estimates the degree to which inflation at the tag transfers to the sequencing SNP ($r_{TS_i} \frac{\delta_{GS_i}}{\sqrt{\delta_G \delta_{S_i}}}$) and adjusts each test statistic accordingly. The quantity $\frac{\delta_{GS_i}}{\sqrt{\delta_G \delta_{S_i}}}$ adjusts the correlation for missing data (see below for details).

The tagging effect is most pronounced when there is low power at the tag SNP. For example, consider the case where we only examine the region if the tag SNP test statistic is significant at level α. Then, using Inverse Mills Ratio, the expected value of the test statistic is

$$Q = E\left[T_{G_{obs}} \middle| T_{G_{obs}} > Z_\alpha\right] = \frac{\phi\left(Z_\alpha - \sqrt{n}\mu_C r_{GC}\right)}{\Phi\left(-\left(Z_\alpha - \sqrt{n}\mu_C r_{GC}\right)\right)}. \qquad \text{Eqn C}$$

This quantity increases when the critical value Z_α is extreme compared to the expected value of the tag SNP test statistic $\sqrt{n}\mu_C r_{GC}$, i.e. when there is low power to detect the tag SNP. The vector of expected values at each test statistic is,

$$\sqrt{n}\mu_C \begin{pmatrix} r_{CS_1} \\ r_{CS_2} \\ \vdots \\ r_{CS_k} \end{pmatrix} + \begin{pmatrix} r_{GS_1} \\ r_{GS_2} \\ \vdots \\ r_{GS_k} \end{pmatrix} Q. \qquad \text{Eqn D}$$

This vector is composed of two terms. The first term is the genetic effect, which maximizes at the causal SNP. The second term is the tagging effect which maximizes at the tag SNP and attenuates as correlation between the tag and sequencing SNPs breaks down. In addition, the second term increases for all SNPs as power at the tag SNP decreases. This second term corresponds to increased probability that SNPs that are in highest LD with the tag achieve the top ranks, regardless of whether they are causal or non-causal.

Genotyping Accuracy Effect

Here we show how genotyping error rates and call rates that vary among SNPs can change the joint distribution of the test statistics so that SNPs with higher error rates are less likely to be top-ranked, regardless of whether they are causal or not. Genotyping algorithms make a trade off between call rates and accuracy by applying quality thresholds [56]. One may reduce a SNP's error rate by filtering out individual calls not meeting stringent quality thresholds; however this increases the missing data rate for that SNP. Since both low accuracy and low call rates lower a SNP's probability of being top-ranked, filtering out low quality calls will not correct for the distortion in the ranking of test statistics. We refer to the combined effect of genotyping error and excluded data as the genotyping accuracy effect.

Now we describe the distribution of the test statistic with genotyping error and missing data. Where $0 < \rho_{Si} < 1$ is the correlation between the true genotype and the observed genotype for sequenced (or imputed) SNP *i*, and δ_i is the call rate (proportion of non-missing genotypes), the distribution of the test statistic using the observed genotypes is

$$T_{S_i} \sim N\left(\rho_{Si}\sqrt{\delta_i n}\,\mu_C r_{CS_i}, 1\right). \qquad \text{Eqn E}$$

The smaller ρ_{Si} and δ_i are, the lower the probability that SNP S_i will be top-ranked, regardless of whether it is causal or non-causal.

Derivation of Equation A

T_C is the normally distributed test statistic for association at the causal SNP. If genotyping accuracy and call rate are perfect then the distribution of T_C is

$$T_C \sim N\left(\sqrt{n}\,\mu_C, 1\right).$$

If the causal SNP is imperfectly captured, and the correlation between the genotypes used to compute test statistic T_C and the actual genotypes at the causal SNP is ρ_C, then the mean of test statistic T is $\sqrt{n}\mu_C\rho_C$ [ref 35, main text]. The Wald test divides the parameter estimate through by its variance, and so the test statistic will still have unit variance.

The genotypes at SNPs in correlation with the causal can be thought of in a similar manner as they imperfectly capture the causal SNP genotypes. Where r_{CG} is the correlation between the causal and tag SNPs, the distribution of the test statistic at the tag SNP is

$$T_G \sim N\left(\sqrt{n}\mu_C r_{CG}, 1\right).$$

Similarly, where r_{CSi} is the correlation between the casual and sequencing SNPs, the distribution of the test statistic at sequencing SNP S_i is

$$T_{S_i} \sim N\left(\sqrt{n}\mu_C r_{CS_i}, 1\right).$$

Where the imputed or sequenced SNP is imperfectly genotyped, and the correlation between the imputed and actual genotypes is ρ_{Si}, then the distribution of the sequencing SNP test statistic is

$$T_{Si} \sim N\left(\sqrt{n}\mu_C r_{CS_i}\rho_{S_i}, 1\right).$$

If correlation between sequencing SNP S_i and the causal SNP C is r_{CS_i} then for the logistic and linear regression case, the correlation between the test statistics is r_{CS_i}. The logistic regression case is derived in Suniel et al 2010. The linear regression case is derived similarly as follows. Consider a quantitative trait y for which the genetic model is

$$y_i = \alpha + \beta_C x_{Ci} + e_i,$$

where y_i is the quantitative trait value for patient i, β_C is the genetic effect at the causal SNP, x_{ci} is the genotype at the causal SNP, α is the intercept and $e_i \sim N\left(0, \sigma^2\right)$ is the normally

distributed error. Let x_{Gi} be the tag SNP genotype for subject i and let the correlation between the tag and causal SNPs be r_{GC}. Let $s_G^2 = \sum x_{Gi}^2$, $s_C^2 = \sum x_{Ci}^2$, $s_{GC} = \sum x_{Gi} x_{Ci}$, and $\hat{r}_{GC} = \frac{s_{GC}}{s_G s_C}$ ($\hat{r}_{GC}$ is the estimate of correlation between tag and causal SNPs while r_{GC} is the actual correlation). Then the covariance between the parameter estimates at the tag and causal SNPs is

$$\text{cov}(\hat{\beta}_C, \hat{\beta}_G) = \text{cov}\left(\frac{\sum x_{Ci} y_i}{\sum x_{Ci}^2}, \frac{\sum x_{Gi} y_i}{\sum x_{Gi}^2} \right) = \sum_i \sum_j \frac{x_{Ci} x_{Gj}}{s_C^2 s_G^2} \text{cov}(y_i, y_j) = \frac{\hat{r}_{GC}}{s_C s_G} \sigma^2 .$$

The variances of the tag and causal SNP estimates are $\text{var}(\hat{\beta}_G) = \sigma^2 / s_G^2$ and $\text{var}(\hat{\beta}_C) = \sigma^2 / s_C^2$.

Let T_G and T_C be the test statistics at the tag and causal SNPs, the correlation between the test statistics is

$$cor(T_C, T_G) = \text{cov}\left(\frac{\hat{\beta}_C}{\hat{\sigma}(\hat{\beta}_C)}, \frac{\hat{\beta}_G}{\hat{\sigma}(\hat{\beta}_G)} \right) \approx \frac{\frac{r_{GC}}{s_C s_G} \sigma^2}{(\sigma / s_C)(\sigma / s_G)} = r_{GC} .$$

Similarly the correlation between the causal SNP and sequencing SNP S_i is $cor(T_C, T_{Si}) \approx r_{CSi}$ and the correlation between the tag SNP and sequencing SNP S_i is $cor(T_G, T_{Si}) \approx r_{GSi}$. If there are k sequencing SNPs, the joint distribution of the tag and sequencing SNPs is Equation A:

$$\begin{pmatrix} T_G \\ T_{S_1} \\ T_{S_2} \\ \vdots \\ T_{S_k} \end{pmatrix} \sim N \left(\sqrt{n} \mu_C \begin{pmatrix} r_{CG} \\ r_{CS_1} \\ r_{CS_2} \\ \vdots \\ r_{CS_k} \end{pmatrix}, \begin{pmatrix} 1 & r_{GS_1} & r_{GS_2} & \cdots & r_{GS_k} \\ r_{GS_1} & 1 & r_{S_1 S_2} & \cdots & r_{S_1 S_k} \\ r_{GS_2} & r_{S_1 S_2} & 1 & \cdots & r_{S_2 S_k} \\ \vdots & \vdots & \vdots & \ddots & \vdots \\ r_{GS_k} & r_{S_1 S_k} & r_{S_2 S_k} & \cdots & 1 \end{pmatrix} \right) .$$

The causal SNP C is one of the sequencing SNPs $S_1 ... S_k$, and for that sequencing SNP r_{CSi} is 1 and r_{GSi} is 1.

Derivation of Equation B

Consider a multivariate normal random variable split into 2 vectors X_1 and X_2.

$$\begin{pmatrix} X_1 \\ X_2 \end{pmatrix} \sim N\left(\begin{pmatrix} \mu_1 \\ \mu_2 \end{pmatrix}, \begin{pmatrix} \Sigma_{11} & \Sigma_{12} \\ \Sigma_{21} & \Sigma_{22} \end{pmatrix}\right).$$

If we condition on $X_2=x_2$ then the distribution of X_1 becomes

$$\left(X_1 \middle| X_2 = x_2\right) \sim N\left(\mu_1 + \Sigma_{12}\Sigma_{22}^{-1}(y_2 - \mu_2), \Sigma_{11} - \Sigma_{12}\Sigma_{22}^{-1}\Sigma_{21}\right).$$

Consider equation A, conditioned on an observed value for the tag SNP test statistic. The conditional distribution for SNP S_i is

$$\left\{\begin{matrix} T_{S_1} \\ T_{S_2} \\ \vdots \\ T_{S_k} \end{matrix} \middle| T_G = T_{G_{obs}} \right\} \sim N\left(\overline{\mu}, \overline{\Sigma}\right)$$

$$\overline{\mu} = \sqrt{n}\mu_C \begin{pmatrix} r_{CS_1} \\ r_{CS_2} \\ \vdots \\ r_{CS_k} \end{pmatrix} + \begin{pmatrix} r_{GS_1} \\ r_{GS_2} \\ \vdots \\ r_{GS_k} \end{pmatrix} \left(T_{Gobs} - \sqrt{n}\mu_C r_{CG}\right)$$

$$\overline{\Sigma} = \begin{pmatrix} 1 - r_{GS_1}^2 & r_{S_1S_2} - r_{GS_1} r_{GS_2} & \cdots & r_{S_1S_k} - r_{GS_1} r_{GS_k} \\ r_{S_1S_2} - r_{GS_1} r_{GS_2} & 1 - r_{GS_2}^2 & \cdots & r_{S_2S_k} - r_{GS2} r_{GS_k} \\ \vdots & \vdots & \ddots & \vdots \\ r_{S_1S_k} - r_{GS_1} r_{GS_k} & r_{S_2S_k} - r_{GS2} r_{GS_k} & \cdots & 1 - r_{GSk}^2 \end{pmatrix}$$

This expression can then be simplified to Equation B:

$$\left\{ \begin{pmatrix} T_{S_1} \\ T_{S_2} \\ \vdots \\ T_{S_k} \end{pmatrix} \mid T_G = T_{G_{obs}} \right\} \sim N\left(\sqrt{n}\mu_C \begin{pmatrix} r_{CS_1} \\ r_{CS_2} \\ \vdots \\ r_{CS_K} \end{pmatrix} + \begin{pmatrix} r_{GS_1} \\ r_{GS_2} \\ \vdots \\ r_{GS_K} \end{pmatrix} (T_{G_{obs}} - r_{GC}\mu_C\sqrt{n}), \Sigma \right), \qquad \text{Eqn B}$$

where the elements of Σ are $\Sigma_{ij} = r_{S_iS_j} - r_{GS_i}r_{GS_j} \quad i = 1...k, j = 1...k$.

Derivation of the joint distribution of the tag and sequencing SNP test statistics

Derivation of the joint distribution of the tag and sequencing SNP test statistics in the presence of imperfect call rates and genotyping inaccuracy is as follows.

If sequencing SNP S_i is observed with no error, and the correlation between the sequencing SNP and causal SNP is r_{CS_i} then the distribution for the sequencing SNP test statistic is

$$T_{S_i} \sim N(\sqrt{n} r_{CS_i} \mu_C, 1).$$

If the sequencing SNP is observed with error and ρ_{S_i} is the correlation between the actual genotypes and the imperfectly called sequenced/imputed genotypes, then the distribution of the test statistic computed using the imperfect sequencing SNP genotypes is

$$T_{S_i} \sim N(\sqrt{n} \rho_{S_i} r_{CS_i} \mu_C, 1).$$

As noted above, misclassification does not affect the variance of the test statistic. If the call rate is less than 100%, Where the call rate is δ_{S_i}, the sample size is effectively scaled by the call rate and the distribution of the test statistic is

$$T_{S_i} \sim N\left(\sqrt{n}\sqrt{\delta_{S_i}} \rho_{S_i} r_{CS_i} \mu_C, 1\right).$$

The tagging effect also depends on the call rate. Consider the extreme case where only ½ of the patients are called at the tag SNP, and the other ½ of the sample is called at sequencing SNP S_i. The sample is effectively divided in two and so selection at the tag SNP has no effect on the test statistic at the sequencing SNP. The correlation between the tag and sequencing SNP test statistics can be worked out as follows.

Consider the quantitative trait model $y_i = \alpha + \beta_C x_{Ci} + e_i$ described above. The covariance between the tag and sequencing SNP test statistics is

$$cor(T_G, T_S) = \frac{\text{cov}(\hat{\beta}_G, \hat{\beta}_S)}{\sigma(\hat{\beta}_G)\sigma(\hat{\beta}_S)},$$

where $\hat{\beta}_G$ is the estimated genetic effect at the tag SNP and $\hat{\beta}_S$ is the estimated genetic effect at sequencing SNP S_i. The covariance between the estimates is

$$\text{cov}(\hat{\beta}_G, \hat{\beta}_S) = \text{cov}\left(\frac{\sum_{j=1}^{n} y_j x_{Tj} d_{Gj}}{\sigma_T^2 \sum d_{Gj}}, \frac{\sum_{j=1}^{n} y_j x_{Sj} d_{Sj}}{\sigma_S^2 \sum d_{Sj}} \right) = \frac{\text{cov}\left(\sum_{j \in A} y_j x_{Gj}, \sum_{j \in A} y_j x_{Sj} \right)}{\sigma_T^2 \sum d_{Gj} \sigma_S^2 \sum d_{Sj}},$$

where σ_T^2 is the variance of the tag SNP genotypes and σ_S^2 is the variance of the sequencing SNP genotypes, indicator variable d_{Sj} is 1 if the sequencing SNP genotype is non-missing for patient i and 0 otherwise, indicator variable d_{Gi} is 1 if the tag SNP genotyped is non-missing for patient j and 0 otherwise. "A" in the summation is the set of all patients j=1...n for whom both the tag and sequencing SNP genotypes are non-missing.

Let δ_G and δ_{S_i} be the proportions of samples with non-missing genotypes (termed call rates) at SNPs G and S_i, respectively, and δ_{GS_i} is the joint call rate, the proportion of samples with non-missing genotypes at both SNPs. Then the covariance between the two estimates is:

$$\operatorname{cov}\left(\hat{\beta}_G, \hat{\beta}_S\right) = \frac{\operatorname{cov}\left(\sum\limits_{j \in A} y_j x_{Gj}, \sum\limits_{j \in A} y_j x_{Sj}\right)}{\sigma_G^2 \sum d_{Gj} \sigma_S^2 \sum d_{Sj}} = \frac{\sum\limits_{j \in A} \operatorname{cov}\left(y_j x_{Gj}, y_j x_{Sj}\right)}{\sigma_G^2 \sigma_S^2 n \delta_G n \delta_S}$$

$$= \frac{\sigma_y^2 \rho_{TS} \sigma_T \sigma_S n \delta_{GS}}{\sigma_G^2 \sigma_S^2 n \delta_G n \delta_S} = \rho_{TS} \frac{\sigma_y^2}{\sigma_G \sigma_S^{\backslash}} \frac{\delta_{GS}}{\delta_G \delta_S} \frac{1}{n} .$$

The double summation in the second expression simplifies to a single summation because $\operatorname{cov}\left(y_i x_{Gi}, y_j x_{Sj}\right) = 0$ where $i \neq j$. The correlation between the test statistics is

$$cor\left(T_G, T_S\right) = \frac{\operatorname{cov}\left(\hat{\beta}_G, \hat{\beta}_S\right)}{\sigma\left(\hat{\beta}_G\right) \sigma\left(\hat{\beta}_S\right)} = \frac{\rho_{TS} \frac{\delta_{GS}}{\delta_G \delta_S} \frac{1}{n}}{\frac{1}{\sqrt{\delta_G n}} \frac{1}{\sqrt{\delta_S n}}} = \rho_{TS} \frac{\delta_{GS}}{\sqrt{\delta_G \delta_S}} .$$

Since the correlation between the test statistics scales the tagging effect we incorporate the term $\frac{\delta_{GS}}{\sqrt{\delta_G \delta_S}}$ into the adjustment in equation (1).

<u>Additional References for Text S2</u>

56. Duitama J, Kennedy J, Dinakar S, Hernández Y, Wu Y, Mandoiu II (2011) Linkage disequilibrium based genotype calling from low-coverage shotgun sequencing reads. *BMC Bioinformatics* 12:S53
