# Supplementary material for: Re-Ranking Sequencing Variants in the Post-GWAS Era for Accurate Causal Variant Identification
Source: PLoS Genet. 2013 Aug 8;9(8):e1003609. doi: 10.1371/journal.pgen.1003609 (PMC3738448; doi:10.1371/journal.pgen.1003609)
Supplement: Text S3 — Low power exacerbates the selection effect. (PDF) [file pgen.1003609.s018.pdf]

Text S3. Low Power Exacerbates the Selection Effect

The selection effect is most pronounced when there is low power at the tag SNP. For example, consider the case where we examine the region only if the tag SNP test statistic is significant at level α (i.e. $T_G \sim N(\sqrt{n}\, r_{CG}\mu_C, 1)$ and $T_G > Z_\alpha$). Then, using the Inverse Mills Ratio [58], the expected bias at the tag SNP is

$$E\left[T_{G_{obs}} \middle| T_{G_{obs}} > Z_\alpha\right] - E\left[T_{G_{obs}}\right] = \frac{\phi(Z_\alpha - \sqrt{n} r_{CG}\mu_C)}{1 - \Phi(Z_\alpha - \sqrt{n} r_{CG}\mu_C)} \text{ ,}$$

where ϕ is the standard normal density function and Φ is the standard normal cumulative distribution function. Here we assume that $E\left[T_{G_{obs}} \middle| |T_{G_{obs}}| > Z_\alpha\right] \approx E\left[T_{G_{obs}} \middle| T_{G_{obs}} > Z_\alpha\right]$, because the tag and causal SNPs are positively correlated and $Pr(T_{G_{obs}} < -Z_\alpha)$ will be very small at genome-wide significance. This bias increases when the critical value Z_α is extreme compared to $\sqrt{n} r_{CG}\mu_C$, the expected value of T_G at the tag SNP, that is when there is low power to detect the tag SNP. In a similar manner, the expected value of T_G conditional on the tag SNP *G* achieving top-rank is inflated.

Additional References for Text S3

58. Heckman JJ (1979) Sample selection bias as a specification error. Econometrica. 47(1): 153-161.
