# Supplementary material for: Re-Ranking Sequencing Variants in the Post-GWAS Era for Accurate Causal Variant Identification
Source: PLoS Genet. 2013 Aug 8;9(8):e1003609. doi: 10.1371/journal.pgen.1003609 (PMC3738448; doi:10.1371/journal.pgen.1003609)
Supplement: Text S4 — Tagging and coverage of GWAS platforms. (PDF) [file pgen.1003609.s019.pdf]

Text S4. Tagging and Coverage of GWAS Platforms

In general, both tagging as well as imputation quality can be lower than investigators expect for the following three reasons.

(1) Manufacturers use the HapMap samples both to design GWAS platforms and to estimate coverage, which results in overestimated coverage rates [59]. Numerous studies have shown that coverage of common variation is substantially lower than reported by microarray manufacturers [60]; this is especially true for non-European samples [61]. Therefore, tagging r^2 is often lower than expected.

(2) Imputation accuracy is not necessarily lower-bounded by tagging r^2. A SNP may be very well tagged, but imputation accuracy depends on how well the model that is derived from the reference panel matches the GWAS sample. For example, if an imputed SNP is perfectly correlated with a tag SNP in the GWAS sample but not in the reference panel, then imputation r^2 can be less than 1. Jiang et al [59] tested the accuracy of imputation in Chinese samples and found that despite Illumina's claim that the OmniExpress chip covers 91% of common SNPs (MAF > 5%) at $r^2 > 0.8$ in Chinese populations, only 73% of common SNPs were imputed at $r^2 > 0.8$.

(3) Sequencing/imputation SNPs are best captured by tag SNPs with similar MAFs, as correlation is bounded when MAFs are unequal. In our simulations, we assume that the tag and sequencing SNPs have similar MAF so that correlation can be allowed to vary from 0.78 to 0.98.

Additional References for Text S4

59. Jiang L, Willner D, Danoy P, Xu H, Brown M (2013) Comparison of the Performance of Two Commercial Genome-Wide Association Study Genotyping Platforms in Han Chinese Samples. *Genes, Genomes Genetics* 3:23-29

60. Bhangale, T. R., Rieder, M. J., & Nickerson, D. A. (2008). Estimating coverage and power for genetic association studies using near-complete variation data. *Nature Genetics*, *40*:841-843

61. Barrett JC & Cardon LR (2006) Evaluating coverage of genome-wide association studies. Nature Genetics 38:659-662
